# Supplementary material for: Docosanoic acid conjugation to siRNA enables functional and safe delivery to skeletal and cardiac muscles
Source: Mol Ther. 2020 Dec 19;29(4):1382–94. doi: 10.1016/j.ymthe.2020.12.023 (PMC8058398; doi:10.1016/j.ymthe.2020.12.023)
Supplement: Document S1. Figures S1–S6 [file mmc1.pdf]

YMTHE, Volume *29*

Supplemental Information

Docosanoic acid conjugation to siRNA enables functional and safe delivery to skeletal and cardiac muscles

Annabelle Biscans, Jillian Caiazzi, Nicholas McHugh, Vignesh Hariharan, Manish Muhuri, and Anastasia Khvorova

A.

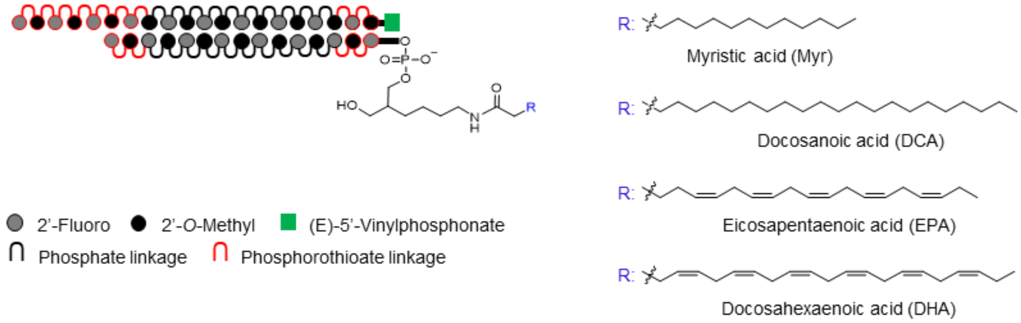

B.

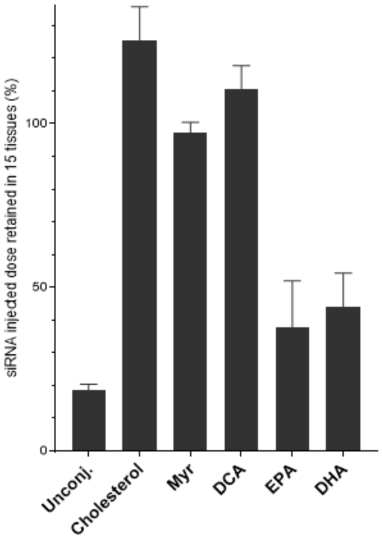

C.

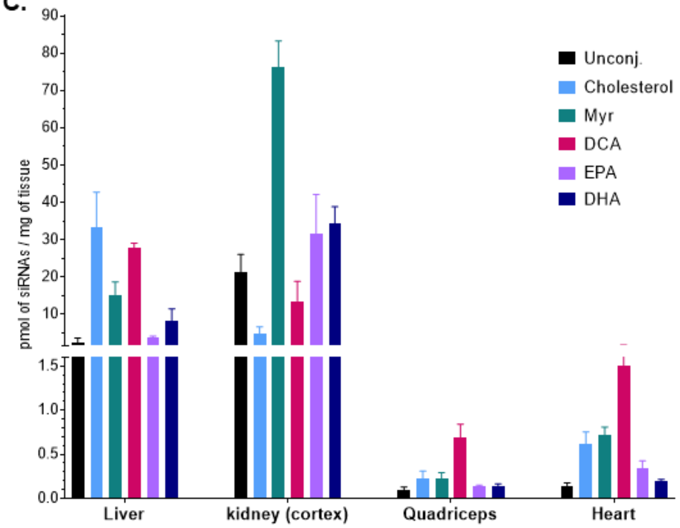

D.

Tissue	Conjugate	% injected dose	Tissue	Conjugate	% injected dose	Tissue	Conjugate	% injected dose	Tissue	Conjugate	% injected dose
Liver	Unconj.	6 ± 3	Kidneys	Unconj.	9 ± 2	Total skeletal muscles	Unconj.	0.6 ± 0.2	Heart	Unconj.	0.03 ± 0.007
	Cholesterol	80 ± 13		Cholesterol	2 ± 1		Cholesterol	1.4 ± 0.5		Cholesterol	0.15 ± 0.020
	Myr	39 ± 8		Myr	29 ± 3		Myr	1.4 ± 0.4		Myr	0.17 ± 0.020
	DCA	71 ± 3		DCA	6 ± 1		DCA	4.3 ± 0.9		DCA	0.40 ± 0.100
	EPA	10 ± 1		EPA	13 ± 3		EPA	1.3 ± 0.8		EPA	0.08 ± 0.020
	DHA	21 ± 8		DHA	13 ± 2		DHA	0.9 ± 0.2		DHA	0.05 ± 0.005

E.

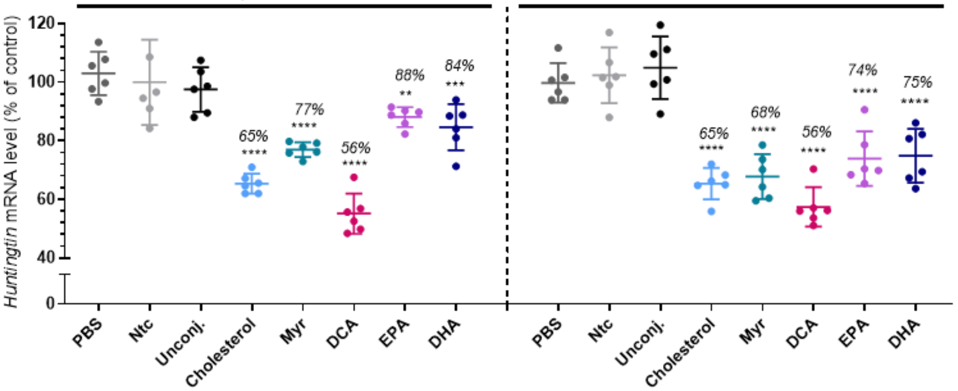

Fig. S1: Previously published data showing DCA conjugate allows quantitative retention in tissues, and significant accumulation and silencing in muscles after a single SC injection. (A.) Schematic representation of fatty acid conjugated siRNAs used to evaluate conjugate impact on muscle delivery (B.) Bar graph showing percent of injected dose retained in tissues at 48h post-injection (average of n = 3 ± SD) (C.) Bar graph showing siRNA quantification in liver, kidneys (cortex), quadriceps, and heart, measured by PNA hybridization assay (48h post-injection, 20 mg/kg, average of n = 3 ± SD) (D.) Table summarizing percent of injected dose retained in liver, kidneys, heart, and muscles at 48h post-injection (average of n= 3 ± SD) (E.) Percent of silencing in quadriceps and heart after SC injection of conjugated siRNA targeting *Huntingtin* (*Htt*) mRNA at 1-week (n=6 mice per group, 20 mg/kg). mRNA levels were measured using QuantiGene® (Affymetrix), normalized to a housekeeping gene, *Hprt* (Hypoxanthine-guanine phosphoribosyl transferase), and presented as percent of PBS (Phosphate buffered saline) control (mean ± SD). Data analysis: Multiple comparisons = One-way ANOVA, Dunnett test (****P<0.0001, ***P<0.001, **P<0.01). ***Data presented in this figure have been reformatted from previous publications (32,33).***

Tissue	Unconj. siRNA	Myr siRNA	DCA siRNA	EPA siRNA	DHA siRNA	Cholesterol siRNA
Liver	2.4 ± 1.3	15.3 ± 3.4	27.9 ± 1.2	3.9 ± 0.3	8.2 ± 3.3	33.3 ± 9.5
kidney (cortex)	21.3 ± 4.7	76.3 ± 7.0	13.5 ± 5.3	31.5 ± 10.6	34.3 ± 4.5	4.7 ± 1.9
Quadriceps	0.1 ± 0.0	0.2 ± 0.1	0.7 ± 0.1	0.1 ± 0.0	0.1 ± 0.0	0.2 ± 0.1
Heart	0.1 ± 0.0	0.7 ± 0.1	1.5 ± 0.3	0.3 ± 0.1	0.2 ± 0.0	0.6 ± 0.1

Fig. S2: DCA conjugate allows significant accumulation in both skeletal and cardiac tissues

Table showing siRNA quantification (pmol of siRNA / mg of tissue) in liver, kidneys (cortex), quadriceps, and heart, measured by PNA hybridization assay (48h post-injection, 20 mg/kg, average of n = 3 ± SD)

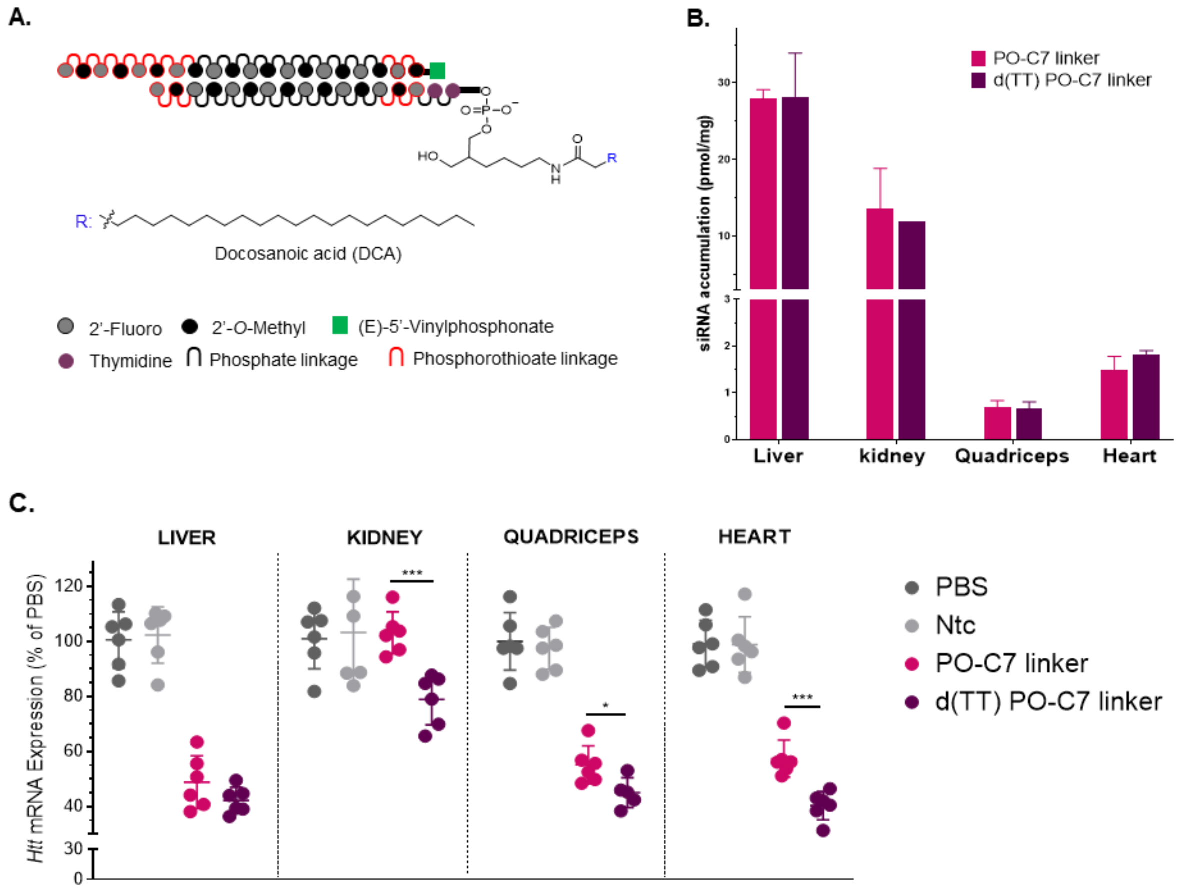

Fig. S3: Previously published data showing d(TT) PO-C7 linker enhanced DCA-conjugated siRNA silencing activity in muscles without affecting tissue accumulation. (A.) Schematic representation of DCA-conjugated siRNAs with d(TT) PO-C7 linker (B.) Bar graph showing siRNA quantification in liver, kidneys (cortex), quadriceps, and heart, measured by PNA hybridization assay (48h post-injection, 20 mg/kg, average of n = 3 ± SD) (C.) Percent of silencing in liver, kidney, quadriceps, and heart after SC injection of DCA siRNA targeting *Huntingtin* (*Htt*) mRNA (n=6 mice per group, 20 mg/kg). mRNA levels were measured using QuantiGene® (Affymetrix), normalized to a housekeeping gene, *Hprt* (Hypoxanthine-guanine phosphoribosyl transferase), and presented as percent of PBS (Phosphate buffered saline) control (mean ± SD). Data analysis: t test (***P<0.001, *P<0.1). ***Data in this figure are derived from (42).***

Tissue	Dose (mg/kg)	Level of accumulation (pmol of siRNA / mg of tissue)	Organ weight (mg)	% injected dose
Liver	20	28 ± 3	1000 (defined experimentally)	71 ± 8
	2*20	47 ± 4		59 ± 5
	6*20	81 ± 8		34 ± 3
	2*20 (1 month)	12 ± 1		15 ± 1
Heart	20	1.4 ± 0.1	94 (defined experimentally)	0.34 ± 0.030
	2*20	1.6 ± 0.2		0.19 ± 0.002
	6*20	1.7 ± 0.1		0.07 ± 0.003
	2*20 (1 month)	0.4 ± 0.04		0.04 ± 0.005
Full body muscles	20	0.4 ± 0.06	2400 (based on publications 53-55)	2.5 ± 0.4
	2*20	0.4 ± 0.04		1.2 ± 0.2
	6*20	0.9 ± 0.05		1.0 ± 0.1
	2*20 (1 month)	0.1 ± 0.03		0.4 ± 0.1

Fig. S4: Levels of tissue accumulation (pmol of siRNA / mg of tissue), and percent of the injected dose retained in liver, heart, and muscles after injection of various doses (20, 2*20 and 6*20 mg/kg), calculated using either experimentally defined organ weights (liver and heart) or based on literature derived organ weights (muscles). Accumulation levels were measured by PNA hybridization assay at 1-week or 1-month post-injection (average of n = 3 ± SD).

Cytokine (pg/mL)	PBS			DCA siRNA 50 mg/kg dose			DCA siRNA 100 mg/kg dose			Chol siRNA 50 mg/kg dose			Chol siRNA 100 mg/kg dose		
	1	2	3	1	2	3	1	2	3	1	2	3	1	2	3
IL-1alpha	13.0	12.6	12.2	12.8	13.4	12.2	15.0	13.4	13.0	12.8	14.6	14.6	19.0	22.1	25.1
IL-1beta	2.8	2.9	3.0	3.0	3.3	2.7	4.0	3.2	2.8	2.7	3.3	2.8	5.2	5.8	6.3
IL-2	3.4	3.5	3.5	8.5	3.5	13.5	4.1	6.8	3.8	3.5	4.0	4.3	5.6	6.0	6.5
IL-3	1.1	1.1	1.1	1.2	1.1	1.2	1.1	1.3	1.2	1.1	1.2	1.1	1.7	2.1	2.4
IL-4	3.1	3.2	3.3	3.3	3.4	3.1	3.9	3.4	3.3	3.3	3.9	3.4	5.9	7.2	8.6
IL-5	7.8	7.8	7.8	9.2	9.2	9.2	10.3	11.8	8.9	7.8	11.6	9.5	17.6	20.7	23.8
IL-6	10.4	10.4	10.4	11.3	11.5	11.0	15.3	17.9	12.7	10.4	15.0	13.3	31.7	31.6	31.4
IL-9	239.0	224.1	209.2	224.1	209.2	239.0	249.0	298.8	229.1	209.2	259.0	239.0	433.3	460.7	488.0
IL-10	18.3	17.5	16.7	17.8	19.3	16.2	23.0	18.3	17.8	17.8	21.4	18.8	31.3	37.4	43.4
IL-12p70	33.6	35.7	37.8	40.0	41.0	38.9	33.0	35.4	37.8	53.6	67.0	69.5	42.2	72.6	103.1
IL-13	5.2	5.0	4.8	5.1	5.0	5.2	5.4	7.3	5.0	5.2	6.3	5.9	10.0	11.6	13.1
IL-15/IL-15R	5.8	5.6	5.4	5.6	5.6	5.6	6.5	6.5	5.6	5.4	6.5	5.8	9.8	11.9	14.0
IL-17A	10.6	10.6	10.6	11.2	11.0	11.4	11.0	11.9	11.4	11.0	11.9	11.0	16.5	17.8	19.1
IL-18	163.2	155.1	146.9	168.7	168.7	168.7	195.9	223.1	179.5	179.5	223.1	195.9	375.4	432.5	489.7
IL-22	49.5	44.1	38.7	46.3	47.4	45.2	58.1	43.0	36.6	40.9	44.1	38.7	75.3	93.1	110.8
IL-23	62.5	61.2	59.8	63.2	65.3	61.2	87.0	59.8	57.1	59.8	62.5	57.1	89.7	107.4	125.1
IL-27	25.0	22.6	20.2	22.6	21.2	24.1	28.9	30.8	19.2	21.2	23.1	21.2	26.0	24.5	23.1
IL-28	129.9	122.1	114.3	128.6	122.1	135.1	142.9	124.7	124.7	129.9	135.1	129.9	223.5	278.1	332.6
IL-31	20.1	20.1	20.1	20.1	20.1	20.1	21.8	23.5	20.9	20.9	21.8	20.9	32.7	36.0	39.4
GM-CSF	4.1	4.0	3.9	3.9	4.1	3.8	4.4	4.6	4.1	4.1	4.4	4.4	7.5	8.6	9.6
M-CSF	0.9	0.9	0.9	1.0	1.0	1.0	0.9	1.4	1.1	1.0	1.2	1.1	2.0	2.4	2.9
G-CSF/CSF-3	8.8	8.9	9.1	9.5	9.8	9.1	11.2	11.9	10.5	9.5	18.9	10.5	61.2	64.9	68.7
IP-10	21.1	19.6	18.0	22.9	21.8	24.1	17.4	25.0	32.6	47.9	78.6	68.7	231.9	231.1	230.2
MCP-1	25.2	27.3	29.4	35.7	36.9	34.4	70.7	87.3	45.3	53.5	113.3	68.8	1363.9	2039.2	2714.4
MCP-3	18.0	19.0	20.0	82.6	88.6	76.5	78.0	105.2	132.3	153.4	333.4	206.8	469.5	473.0	476.5
MIP-1alpha	2.0	1.9	1.9	1.9	1.9	1.9	2.2	2.4	2.2	1.9	2.4	2.4	5.9	6.5	7.2
MIP-beta	3.0	3.0	3.0	3.1	3.0	3.2	4.2	5.4	4.6	4.2	5.3	5.8	38.4	36.3	34.3
MIP-2	3.5	3.5	3.5	3.9	3.9	4.0	3.6	5.3	3.8	3.9	4.7	6.5	6.8	7.3	7.9
Eotaxin	483.6	479.9	476.1	484.3	493.7	474.8	472.5	829.3	705.8	617.7	825.2	839.5	1181.0	1158.2	1135.4
RANTES	20.7	20.4	20.0	17.7	19.0	16.5	20.6	29.5	22.0	17.7	21.5	23.8	30.5	34.0	37.5
Gro-alpha/KC	19.2	17.3	15.4	20.0	25.1	14.9	34.4	53.1	15.6	21.8	30.8	17.5	65.9	69.4	73.0
IFN-alpha	25.6	28.0	30.5	34.4	43.0	25.7	35.9	33.9	31.8	23.1	35.7	21.1	83.3	96.5	109.7
IFN-gamma	3.3	3.2	3.1	3.3	3.1	3.4	3.5	4.0	3.4	3.3	3.6	3.4	4.7	5.4	6.1
TNF-alpha	10.7	11.0	11.2	11.0	10.3	11.7	23.3	26.6	13.1	11.7	17.7	11.0	30.4	29.7	29.0

Fig. S5: DCA conjugate does not induce cytokine elevation at both doses where cholesterol showed significant toxicity at high dose. Heat map showing cytokine concentrations (pg/mL) in serum at 24h after SC injections of DCA- and cholesterol (chol)-conjugated siRNAs at 50 and 100 mg/kg (n=3 mice per group, 1, 2 and 3 represent each mouse).

Cytokine (pg/mL)	PBS			DCA siRNA 20 mg/kg dose			DCA siRNA 2*20 mg/kg dose			DCA siRNA 6*20 mg/kg dose		
	1	2	3	1	2	3	1	2	3	1	2	3
G-CSF	10.9	12.4	13.3	9.9	12.6	12.8	10.9	9.3	10.7	10.7	11.4	12.8
GRO alpha	24.1	33.4	45.8	9.5	51.9	25.7	22.6	31.3	23.6	14.7	36.3	30.3
IFN gamma	6.0	5.8	6.0	5.3	5.7	6.2	5.9	5.8	5.3	6.2	6.1	6.8
IL-12p70	15.9	16.9	17.9	15.4	17.6	18.6	15.9	15.4	15.4	16.9	16.4	17.4
IL-6	15.5	15.5	17.9	14.4	19.1	19.1	15.5	15.5	15.5	15.5	16.7	19.1
IP-10	24.4	24.7	28.0	12.0	20.4	33.8	21.3	20.5	16.6	23.5	38.9	36.4
M-CSF	0.6	0.6	0.6	0.5	0.6	0.8	0.5	0.5	0.4	0.5	0.6	0.6
MCP-1	15.6	18.5	21.4	13.9	17.3	17.3	18.5	17.3	15.6	20.8	19.6	15.6
MCP-3	14.2	14.1	10.2	13.7	8.1	15.7	21.5	11.6	7.9	30.3	34.5	29.8
MIP-1beta	0.8	0.9	1.3	0.8	0.8	0.9	0.9	0.9	0.8	1.0	1.0	1.1
TNF alpha	10.3	11.0	14.1	7.5	10.8	9.7	9.1	8.8	8.1	10.3	8.1	10.3

Fig. S6: DCA conjugate does not induce cytokine elevation after multiple injections. Heat map showing cytokine concentrations (pg/mL) in serum at 1 week after SC injections of DCA-conjugated siRNAs at 20, 2*20, and 6*20 mg/kg (n=3 mice per group, 1, 2 and 3 represent each mouse).
