# Supplementary material for: Docosanoic acid conjugation to siRNA enables functional and safe delivery to skeletal and cardiac muscles
Source: Mol Ther. 2020 Dec 19;29(4):1382–94. doi: 10.1016/j.ymthe.2020.12.023 (PMC8058398; doi:10.1016/j.ymthe.2020.12.023)
Supplement: Document S2. Article plus supplemental information [file mmc2.pdf]

Molecular Therapy
Original Article

Docosanoic acid conjugation to siRNA enables functional and safe delivery to skeletal and cardiac muscles

Annabelle Biscans,[1,2] Jillian Caiazzi,[1,2] Nicholas McHugh,[1,2] Vignesh Hariharan,[1,2] Manish Muhuri,[3,4,5] and Anastasia Khvorova[1,2]

[1]RNA Therapeutics Institute, University of Massachusetts Medical School, Worcester, MA 01604, USA; [2]Program in Molecular Medicine, University of Massachusetts Medical School, Worcester, MA 01604, USA; [3]Horae Gene Therapy Center, University of Massachusetts Medical School, Worcester, MA 01604, USA; [4]Department of Microbiology and Physiological Systems, University of Massachusetts Medical School, Worcester, MA 01604, USA; [5]VIDE Program, University of Massachusetts Medical School, Worcester, MA 01604, USA

Oligonucleotide therapeutics hold promise for the treatment of muscle- and heart-related diseases. However, oligonucleotide delivery across the continuous endothelium of muscle tissue is challenging. Here, we demonstrate that docosanoic acid (DCA) conjugation of small interfering RNAs (siRNAs) enables efficient (~5% of injected dose), sustainable (>1 month), and non-toxic (no cytokine induction at 100 mg/kg) gene silencing in both skeletal and cardiac muscles after systemic injection. When designed to target *myostatin* (muscle growth regulation gene), siRNAs induced ~55% silencing in various muscle tissues and 80% silencing in heart, translating into a ~50% increase in muscle volume within 1 week. Our study identifies compounds for RNAi-based modulation of gene expression in skeletal and cardiac muscles, paving the way for both functional genomics studies and therapeutic gene modulation in muscle and heart.

INTRODUCTION

Therapeutic oligonucleotides have the potential to revolutionize medicine because of their potency, duration of effect, and ability to target previously "undruggable" disease genes.[1–8] The clinical success of oligonucleotides is dependent on their efficient delivery to disease tissues, which is achieved through full chemical stabilization and conjugation.[4,9–19] The trivalent *N*-acetylgalactosamine (GalNAc) conjugate, which binds hepatocyte-specific receptors, has dominated the development of oligonucleotide therapeutics to treat liver diseases, recently demonstrated by the US Food and Drug Administration (FDA) approval of givosiran.[20–24] A single subcutaneous administration of GalNAc-conjugated small interfering RNAs (siRNAs) can induce a clinical benefit that lasts up to 6–12 months in humans.[8,25] The success of the GalNAc conjugate platform demonstrates that functional tissue delivery of therapeutic oligonucleotides is the foundation for any clinical exploration.

Oligonucleotide therapeutics holds promise for the treatment of muscle-related diseases.[26,27] When injected locally, oligonucleotides can achieve significant target gene reduction in a small portion of muscle tissue,[28–30] but its limited distribution minimizes potential therapeutic use. When injected systemically, oligonucleotides (and the majority of other drugs) naturally accumulate in liver, which is a primary filtering tissue with high blood flow volumes and discontinued fenestrated epithelia.[31] Thus, systemic delivery of oligonucleotides to extrahepatic tissues, like muscles, remains a challenge.

Lipid conjugation, such as cholesterol and fatty acids, significantly improves the systemic delivery of oligonucleotides to tissues beyond liver[9,32–36] and supports productive silencing in these extrahepatic tissues.[32,33,37,38] However, only limited success in delivering oligonucleotides to muscle after systemic administration has been reported.[37–39] In the context of siRNAs, only cholesterol conjugation has been evaluated. Although cholesterol-conjugated siRNAs do deliver to muscles after intravenous injection,[38] a high dose (50 mg/kg) is required to achieve sustainable gene silencing. Cholesterol conjugates are highly toxic at high concentrations, limiting their potential for clinical translation.[39]

Fatty acids are involved in the contractile work of skeletal and cardiac cells and are efficiently transported across the muscular endothelium barrier to reach muscle cells,[40] making them potentially viable candidates for conjugate-mediated oligonucleotide delivery to muscle. Indeed, efficient distribution of antisense oligonucleotides (ASOs) to muscle has already been achieved by conjugating ASOs with fatty acids.[37] Palmitic acid, in particular, allowed for robust silencing in muscles after systemic injection.[39,41] For siRNAs, we previously evaluated the impact of fatty acid conjugation on distribution and silencing activity in a variety of tissues, including muscles. Several conjugates (e.g., cholesterol, myristic acid [Myr], docosanoic acid [DCA], eicosapentaenoic acid [EPA], and docosahexanoic acid

Received 12 September 2020; accepted 15 December 2020;
https://doi.org/10.1016/j.ymthe.2020.12.023.

Correspondence: Anastasia Khvorova, RNA Therapeutics Institute, University of Massachusetts Medical School, Worcester, MA 01604, USA.
E-mail: anastasia.khvorova@umassmed.edu

 © 2020 The Author(s).
This is an open access article under the CC BY license (http://creativecommons.org/licenses/by/4.0/).

[DHA]) enabled wide distribution profiles,[32,33] with DCA variants showing enhanced cardiac and skeletal muscle delivery after systemic administration in mice. We further optimized the chemical structure and linker chemistry of DCA-siRNA to enhance productive silencing in extrahepatic tissues.[42]

Here, we evaluated the potential of the optimized DCA-conjugated siRNA scaffold to silence a therapeutically relevant gene, *myostatin*, in muscle and heart. DCA-siRNAs demonstrated productive (~55%–80%) silencing, which lasts longer than 1 month, translating into a ~50% increase in muscle volume. Furthermore, an exaggerated pharmacology study showed a lack of significant cytokine induction at a high dose (100 mg/kg), demonstrating the therapeutic potential of DCA-conjugated siRNAs.

RESULTS

Previous identification of an optimized conjugate structure and siRNA architecture for efficient delivery of siRNAs to muscle

In our previous reports, we synthesized a panel of siRNAs conjugated with saturated and unsaturated fatty acids of varying carbon chain length and unsaturation, i.e., Myr (14:0), DCA (22:0), EPA (20:5 n-3), and DHA (22:6 n-3), and evaluated the impact on relative tissue distribution in mice. Each lipid conjugate was attached to the 3′ end of the siRNA sense strand, which tolerates a range of covalent modifications .[7,39,40] siRNAs were fully chemically modified for maximal stability and minimal innate immune activation.[11–14,17–19,43] Figure S1 shows the cumulative data from these previous reports[32,33] in muscle, heart, and major clearance tissues (liver, kidney).

Approximately 85% of unconjugated siRNAs (control) were cleared from the body (Figure S1B), with retained compounds accumulating primarily in kidneys. Fatty acid conjugation enhanced overall compound retention in a structure-dependent manner: 40%–45% of unsaturated fatty acid siRNAs (EPA and DHA) were retained, whereas quantitative (~100%) retention was observed with cholesterol (control) and saturated fatty acid siRNAs (Myr and DCA) (Figure S1B). The structure of the conjugate also significantly affected tissue distribution (Figures S1C and S2). Cholesterol and a long, saturated chain (DCA) conjugation led to higher siRNA accumulation in liver (33 and 28 pmol/mg, respectively) than in kidneys (5 and 13 pmol/mg, respectively). By contrast, siRNAs conjugated with a short, saturated chain (Myr) or unsaturated chains (EPA and DHA) showed higher accumulation in kidneys (76, 32, and 34 pmol/mg, respectively) than in liver (15, 4, and 8 pmol/mg, respectively). Although the majority of injected siRNAs accumulate in liver and kidneys, a small fraction of compounds was retained in muscle tissues (up to 4.3% of injected dose in skeletal muscles and 0.4% in heart) (Figure S1D). DCA siRNAs distributed to muscle tissues significantly more than other siRNAs, e.g., 3-fold and 2.5-fold higher accumulation in skeletal muscle and heart, respectively, compared with cholesterol-siRNAs (Figure S1C). The difference in accumulation was specific to DCA, because changing both length (Myr) and degree of saturation (EPA and DHA) had a negative impact on siRNA accumulation in muscle. The increase in accumulation translated into functional gene silencing (of *huntingtin* mRNA) by DCA-conjugated siRNA in both skeletal and cardiac muscles (up to 44% silencing; Figure S1E).[32] Our previous reports demonstrate that conjugate chemical structure, which defines serum protein binding and clearance kinetics,[33,34,37,41,44–46] drives tissue distribution, and that DCA conjugation may be optimal for siRNA delivery to muscle.

In all experiments reported in Figure S1 and other publications,[32,33] lipid conjugates are directly attached to siRNAs using a phosphodiester carbon linker (PO-C7 linker) (Figure S1A). In our most recent report,[42] we evaluated the impact of linker chemistry on DCA-siRNA efficacy and found that using a cleavable linker, d(TT) PO-C7 (Figure S3A), significantly improves *huntingtin* mRNA silencing in quadriceps (by 10%, $p < 0.05$) and heart (by 19%, $p < 0.001$) without altering tissue accumulation (Figures S3B and S3C). This is likely because the phosphodiester d(TT) linker has limited *in vivo* stability, sufficient to support initial tissue distribution, but gets quickly degraded upon cellular uptake to allow siRNA release from the conjugate.[47] Collectively, our previous reports identify an optimal conjugated siRNA platform for improving delivery to muscle. In the current study, we set out to apply our optimal siRNA architecture to silence a therapeutically relevant gene in muscle.

Experimental design to evaluate dosing and efficacy of d(TT) PO-C7-linked DCA-conjugated siRNAs targeting a therapeutically relevant gene, *myostatin*, in cardiac and skeletal muscles of mice

The muscle growth factor, myostatin (*Mstn*), is emerging as a therapeutic target of interest for the prevention of muscle wasting.[48] *Mstn* (also known as Growth and Differentiation Factor 8 [GDF-8]) negatively regulates muscle mass and is primarily expressed in skeletal muscles, with low mRNA levels also reported in cardiac tissues.[49] *Mstn* inhibition is associated with increased muscle mass.[50,51] We designed d(TT) PO-C7-linked DCA-conjugated siRNAs to silence *Mstn* using sequences extracted from Khan et al.[38] To better understand the impact of dose on compound accumulation and efficacy in heart and muscles, we injected mice (n = 6 per group) subcutaneously (s.c.) with a range of DCA-siRNA doses: a single dose at 20 mg/kg ("20"), two doses of 20 mg/kg given 10 h apart ("2 × 20"), or six doses administrated over 3 days (morning and night, ~10–14 h apart, "6 × 20"). PBS and a non-targeting siRNA (*Ntc*, compound of identical chemical configuration, but not targeting *Mstn* mRNA) were used as controls for expression and muscle phenotype analysis. We intentionally dosed within short periods of time to explore whether saturation of the primary clearance tissues (liver/kidney) may allow for better siRNA delivery to muscles. At 1 week and 1 month post-injection, we measured siRNA tissue accumulation and Mstn expression (mRNA and protein). To evaluate the effect of DCA-siRNA-mediated *Mstn* inhibition on muscle growth, we also measured muscle size/weight at 1 week and 1 month post-injection.

siRNA dosing regimen must be optimized separately for muscle and heart delivery

To evaluate siRNA tissue accumulation, which is predictive of duration of effect,[11,13,52] we quantified antisense strands in liver, heart,

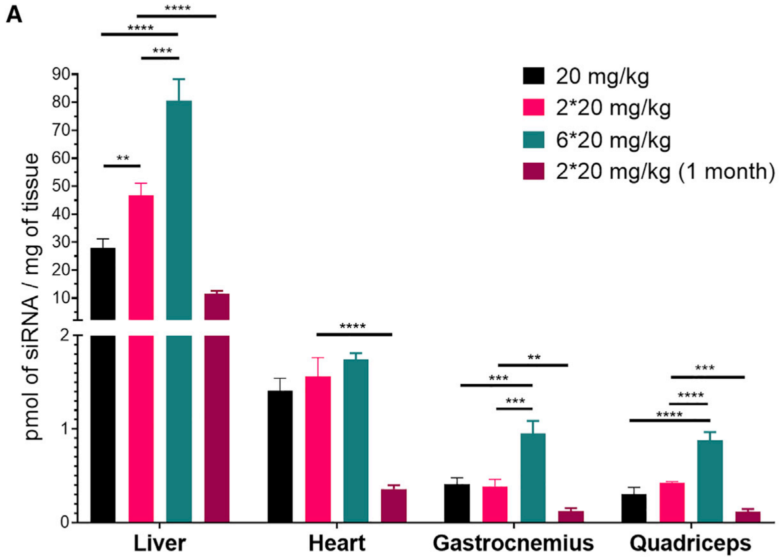

B

Tissue	Dose (mg/kg)	% injected dose*
Liver	20	71 ± 8
	2*20	59 ± 5
	6*20	34 ± 3
	2*20 (1 month)	15 ± 1
Heart	20	0.34 ± 0.030
	2*20	0.19 ± 0.002
	6*20	0.07 ± 0.003
	2*20 (1 month)	0.04 ± 0.005
Full body muscles	20	2.5 ± 0.4
	2*20	1.2 ± 0.2
	6*20	1.0 ± 0.1
	2*20 (1 month)	0.4 ± 0.1

*The relative mass of each tissue has been experimentally defined (liver and heart), or approximated based on published mouse organ weights (full body muscles). Liver 1000g, Heart 94mg and muscles 2400mg

Figure 1. DCA conjugate supports significant accumulation in muscle tissues after s.c. injection at 1 week and 1 month post-injection

(A) Bar graph showing siRNA quantification after 1 week (20, 2 × 20, and 6 × 20 mg/kg) and 1 month (2 × 20 mg/kg 1 month) post-injection in liver, heart, gastrocnemius, and quadriceps, measured by PNA hybridization assay (average of n = 6 ± SD). Data analysis: t test (****$p < 0.0001$, ***$p < 0.001$, **$p < 0.01$). (B) Table summarizing percent of injected dose retained in liver, heart, and muscles (average of 6 mice ± SD).

gastrocnemius muscle, and quadriceps at either 1 week or 1 month post-injection using the peptide nucleic acid (PNA) hybridization assay (see Materials and methods) (Figure 1A). siRNA accumulation in tissues is independent of the target, but it is mainly driven by the chemical composition and conjugate.[32,34,42] Therefore, the evaluation of the distribution of a non-targeting DCA-conjugated siRNA was not necessary as control because it has a similar distribution of targeting DCA-conjugated siRNA, where the conjugate DCA drives distribution and level of accumulation. To estimate the fraction of the injected dose retained in different tissues (Figure 1B), we based the calculations on either the actual weight of the organs when experimentally measurable (for liver and heart) or on published mouse organ weights (for total muscle weight) corresponding to the same mouse strain, sex, and age.[53–55] Consistent with our previous report, DCA-conjugated siRNAs accumulated preferentially in liver (Figure 1; Figure S4), but a significant fraction of the injected dose accumulated in cardiac (up to 0.3%) and skeletal (up to 2.5%) muscles (Figure 1B). The compounds distributed to the same extent in various skeletal muscle types (gastrocnemius and quadriceps), demonstrating that systemic administration of the compounds enabled uniform distribution throughout all muscle tissues, which is important for eventual therapeutic translation.

Changing the dosing regimen had a profoundly different impact on siRNA accumulation in the tissues tested. At 1 week post-injection, two doses within 10 h led to a ~2-fold increase in compound accumulation in liver compared with a single dose (20 versus 2 × 20 mg/kg) (Figure 1A), suggesting that the liver was not saturated after the first injection. However, four additional doses in the next 48 h (6 × 20 mg/kg) led to only a 1.7-fold increase in accumulation (2 × 20 versus 6 × 20 mg/kg), and a large fraction of the compounds got cleared (~65% of the injected dose; Figure 1B). Thus, two injections at 20 mg/kg within 10 h were sufficient to temporarily saturate the liver.

In skeletal muscles, the opposite trend was observed. No significant change in accumulation was detected after one versus two injections (~0.4 versus 0.39 pmol/mg; Figure 1B), suggesting that, at ~10 h, muscle tissues were still saturated with siRNAs from the first dose (Figure 1A). However, additional doses within the next 48 h enabled a ~2.5-fold (0.95 pmol/mg) increase in accumulation. These results indicate that doses need to be at least 1 day apart to enable additional siRNA accumulation in skeletal muscles. Interestingly, in heart, changing the dosing regimen had no significant impact on compound accumulation. One, two, or six injections within 3 days led to a similar level of compound accumulation (~1.4, ~1.6, and ~1.7 pmol/mg, respectively; ~8% increase) (Figure 1A), indicating that a single dose was sufficient to saturate heart tissue. It is likely that a longer gap between the doses (>3 days) would be necessary to enhance siRNA accumulation in the heart. Collectively, these results demonstrate that dosing regimen and dosing time period need to be defined and optimized separately for different tissues to maximize compound tissue accumulation.

At 1 month post-injection, a decrease in compound accumulation was observed in all tissues (~4-fold decrease compared with 1-week accumulation) at the 2 × 20 mg/kg dose (Figure 1A). However, a significant level of siRNAs was still detectable in heart and skeletal muscles (0.36 and 0.12 pmol/mg, respectively), showing that DCA-conjugated fully chemically stabilized siRNAs achieve sustained muscle and heart accumulation.

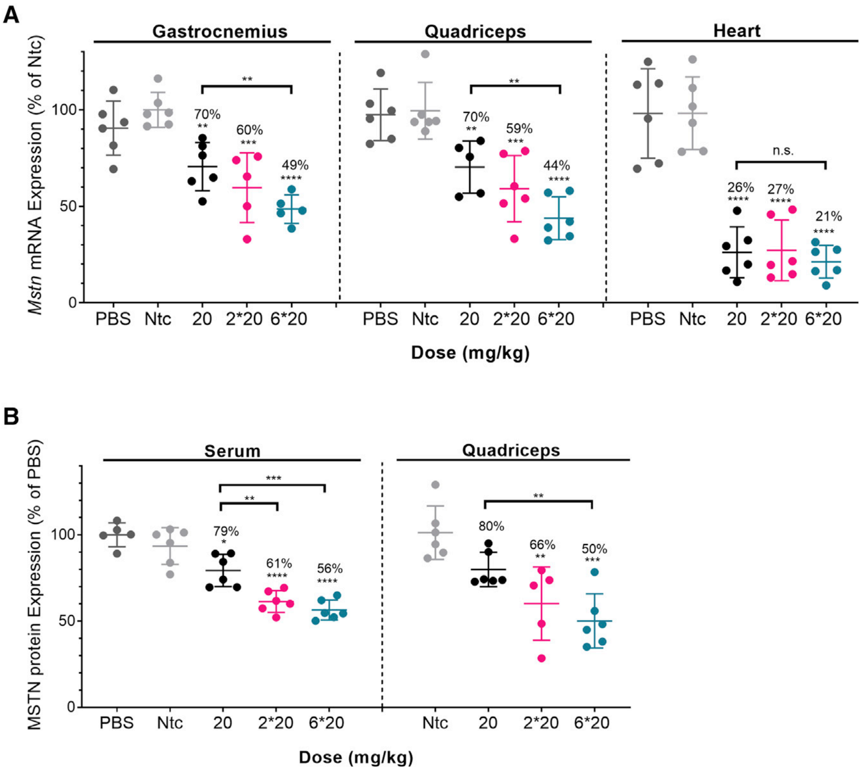

Figure 2. Significant silencing of *Mstn* mRNA and MSTN protein in muscles is achieved using DCA-conjugated siRNAs

(A) Percent of *Mstn* mRNA silencing in gastrocnemius, quadriceps, and heart after s.c. injection of DCA-conjugated siRNA in mice sacrificed at 1 week post-injection (n = 6 mice per group; 20, 2 × 20, and 6 × 20 mg/kg). mRNA levels were measured using QuantiGene (Affymetrix), normalized to a housekeeping gene, Hprt (hypoxanthine-guanine phosphoribosyl transferase), and presented as percent of Ntc (mean ± SD). (B) Percent of MSTN protein levels in serum and quadriceps. s.c. injection of DCA-conjugated siRNA in mice sacrificed at 1 week post-injection (n = 6 mice per group; 20, 2 × 20, and 6 × 20 mg/kg). Protein levels were measured using GDF-8/MSTN Quantikine ELISA kit (R&D Systems), presented as percent of PBS. Data analysis: multiple comparisons = one-way ANOVA, Dunnett test (****p < 0.0001, ***p < 0.001, **p < 0.01, *p < 0.1). n.s., non-significant.

d(TT) PO-C7-linked DCA-siRNAs induce sustained silencing of *Mstn* mRNA in muscles and reduced Mstn protein levels

To evaluate whether the observed levels of siRNA accumulation correlated with observed silencing, we measured *Mstn* expression in heart, gastrocnemius, and quadriceps muscle at either 1 week or 1 month post-injection (Figures 2 and 3). Non-targeting controls (*Ntc*) (a compound of identical chemical configuration and DCA conjugated) showed no significant reduction in target gene expression, indicating that the observed silencing is due to sequence-specific effects, not the general siRNA chemical scaffold. For all doses tested, significant silencing of *Mstn* mRNA was achieved (Figure 2A). In skeletal muscles (gastrocnemius and quadriceps), a dose-dependent reduction in *Mstn* mRNA levels was observed: one injection induced 30% silencing ($p < 0.01$), two injections induced 40% silencing ($p < 0.001$), and six injections induced 51%–56% silencing ($p < 0.0001$). Although there was a positive trend between higher accumulation and higher silencing efficacy, the observed correlation did not reach statistical significance. For example, injecting six doses (6 × 20 mg/kg) versus two doses (2 × 20 mg/kg) of siRNA resulted in a ~2.5-fold increase ($p < 0.0002$) in accumulation (Figure 1) but no significant difference in observed silencing (51%–56% versus 40%–41%; Figure 2A). Thus, although higher accumulation translates toward longer duration of effect, a large number of repeated doses was not necessary, and two doses were sufficient to induce significant activity in skeletal muscles. In cardiac tissue, all doses tested led to similar accumulation (Figure 1; Figure S4) and induced similar silencing (73%–79% silencing; $p < 0.0001$; Figure 2A), with no significant difference compared with the single dose.

To evaluate the duration of observed silencing, which is critical for potential clinical applications, we evaluated *Mstn* mRNA expression in tissues 1 month post-injection (2 × 20 mg/kg 1 month) (Figure 3A). Two doses were s.c. administrated because this dose regimen was sufficient to induce significant silencing at 1 week post-injection. We observed robust silencing in both skeletal and cardiac muscle tissues. Most importantly, there was no statistically significant difference in silencing between 1 week and 1 month post-injection: 40% versus 38% in gastrocnemius muscle, 41% versus 43% in quadriceps, and 73% versus 65% in heart (Figure 3A). These results indicate that s.c. injection of DCA-conjugated siRNAs induces sustained (at least 1 month) gene silencing. Thus, DCA conjugation represents a viable platform for reducing gene expression in muscles and heart.

Reduced *Mstn* mRNA levels correlated with reductions in serum and muscle Mstn protein. A higher dose (6 × 20 mg/kg) induced better protein silencing (44% in serum and 53% in quadriceps; Figure 2B). Because serum level is an indicator of cumulative myostatin expression in the body as a whole, a reduction in MSTN in serum is indicative that there is an overall loss of Mstn silencing. Similarly, at 1 month post-injection, significant Mstn protein silencing was still observed (Figure 3B), confirming the long-term activity of the siRNA compound.

Silencing Mstn in muscles using DCA-conjugated siRNAs leads to a significant increase in muscle volume

For all doses tested, we observed a significant increase in size of skeletal muscles compared with the control groups (Figure 4A).

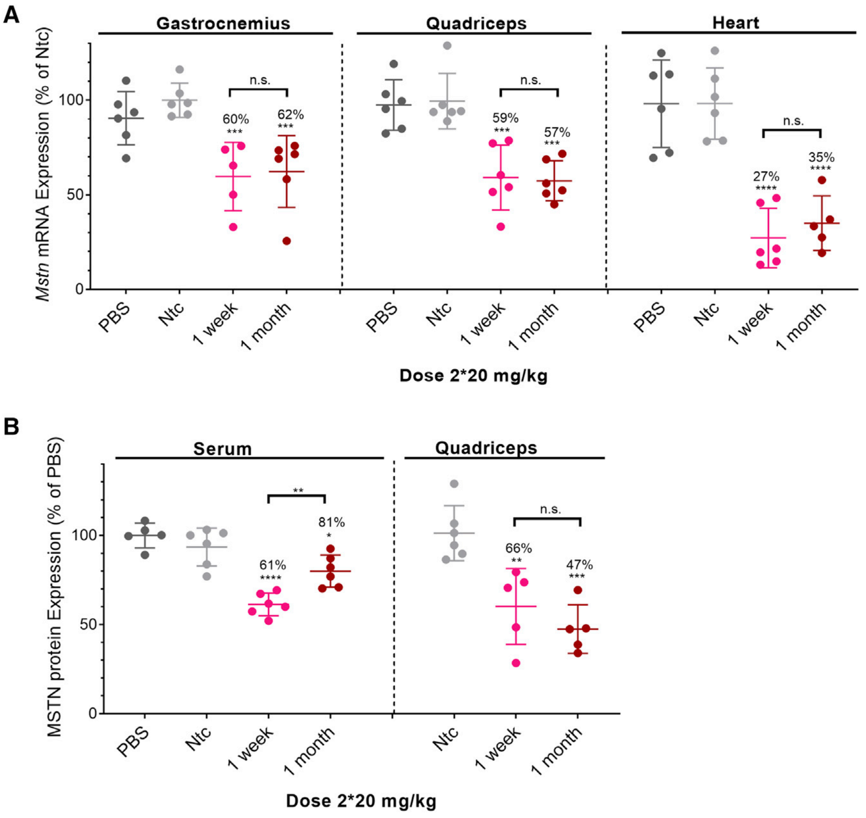

Figure 3. Long-term Mstn mRNA and protein silencing in muscles is achieved using DCA-conjugated siRNAs

(A) Percent of *Mstn* mRNA silencing in gastrocnemius, quadriceps, and heart after s.c. injection of DCA-conjugated siRNA in mice sacrificed at 1 week and 1 month post-injection (n = 6 mice per group; 2 × 20 mg/kg). mRNA levels were measured using QuantiGene (Affymetrix), normalized to a housekeeping gene, Hprt, and presented as percent of Ntc (mean ± SD). (B) Percent of MSTN protein levels in serum and quadriceps. s.c. injection of DCA-conjugated siRNA in mice sacrificed at 1 week and 1 month post-injection (n = 6 mice per group, 2 × 20 mg/kg). Protein levels were measured using GDF-8/MSTN Quantikine ELISA kit (R&D Systems), presented as percent of PBS. Data analysis: multiple comparisons = one-way ANOVA, Dunnett test (**p < 0.01).

The enhancement of thigh size correlated with the administered dose and the silencing observed in muscle tissues (Figure 2A). At 1 week post-injection, the lower dose (20 mg/kg) led to an increase in muscle size of 20%, the intermediate dose (2 × 20 mg/kg) led to an increase of 30%, and the higher dose (6 × 20 mg/kg) led to an increase of 50%. Remarkably, at 1 month post-injection, the increase in thigh size was still maintained (30% compared with controls) and was similar to the thigh size measured at 1 week post-injection with the same dose injected (2 × 20 mg/kg). The improvement in muscle volume was also supported by visual observation of the mice, showing a major increase in leg muscle size (images in Figure 4A).

The improvement in muscle size translated to an increase in total body weight by 6%–8% when compared with PBS groups (Figure 4B). The difference in body weight was observable as early as day 4 after the last injection, which may correlate with the beginning of *Mstn* gene inhibition and fiber muscle improvement. In addition to skeletal muscle growth, we observed a significant enhancement of heart weight when mice were treated with DCA compounds (up to 11% increase) (Figure 4C). However, when normalized to body weight, there was no significant change compared with the PBS group (<0.3%), suggesting a lack of cardiac hypertrophy. Collectively, these results demonstrate a long duration of effect to induce muscle growth and validate the potential of DCA-conjugated siRNAs to achieve clinically relevant outcomes.

DCA-conjugated siRNAs do not induce cytokine elevation

Dose-limiting toxicity of highly chemically modified oligonucleotides has been observed, restraining their potential clinical translation.[56] To evaluate the toxicity of DCA-conjugated siRNAs and determine whether DCA can be used safely, we measured cytokine levels in mice (n = 3 per group) after s.c. injection of compounds at 50 mg/kg and at a high dose of 100 mg/kg. As a reference, cholesterol-conjugated siRNAs were also injected at the same doses. A large panel (34 in total) of cytokines was evaluated, including interleukins, colony-stimulating factors, chemokines, and interferons (Figure 5; Figure S5).

As expected, a high dose (100 mg/kg) of cholesterol-conjugated siRNAs significantly elevated all cytokine levels. Increases of 2- to 3-fold in interleukin, 2- to 7-fold in colony-stimulating factor, 3- to 80-fold in chemokine, and 2- to 3-fold in interferon concentrations were observed compared with PBS, and the elevation of 33 out of 34 cytokines reached statistical significance (Figure 5). At the lower dose (50 mg/kg), cholesterol was tolerated and did not generate significant increases in cytokine levels, except for the chemokines IP10 ($p < 0.001$) and MCP-3 ($p < 0.001$). These results confirmed that cholesterol conjugation is not a viable therapeutic paradigm for systemic delivery due to its dose-limiting toxicity and limited therapeutic index. Remarkably, at both doses (50 and 100 mg/kg), DCA-conjugated compounds did not induce any statistically significant enhancement in cytokine levels. These results demonstrate that DCA is significantly safer than cholesterol and may have potential as a therapeutic for muscle-related diseases.

In addition, even if after a single injection at high doses (100 mg/kg) DCA-conjugated siRNAs do not induce significant cytokine elevation, repetitive injections at lower doses (as reported in this study)

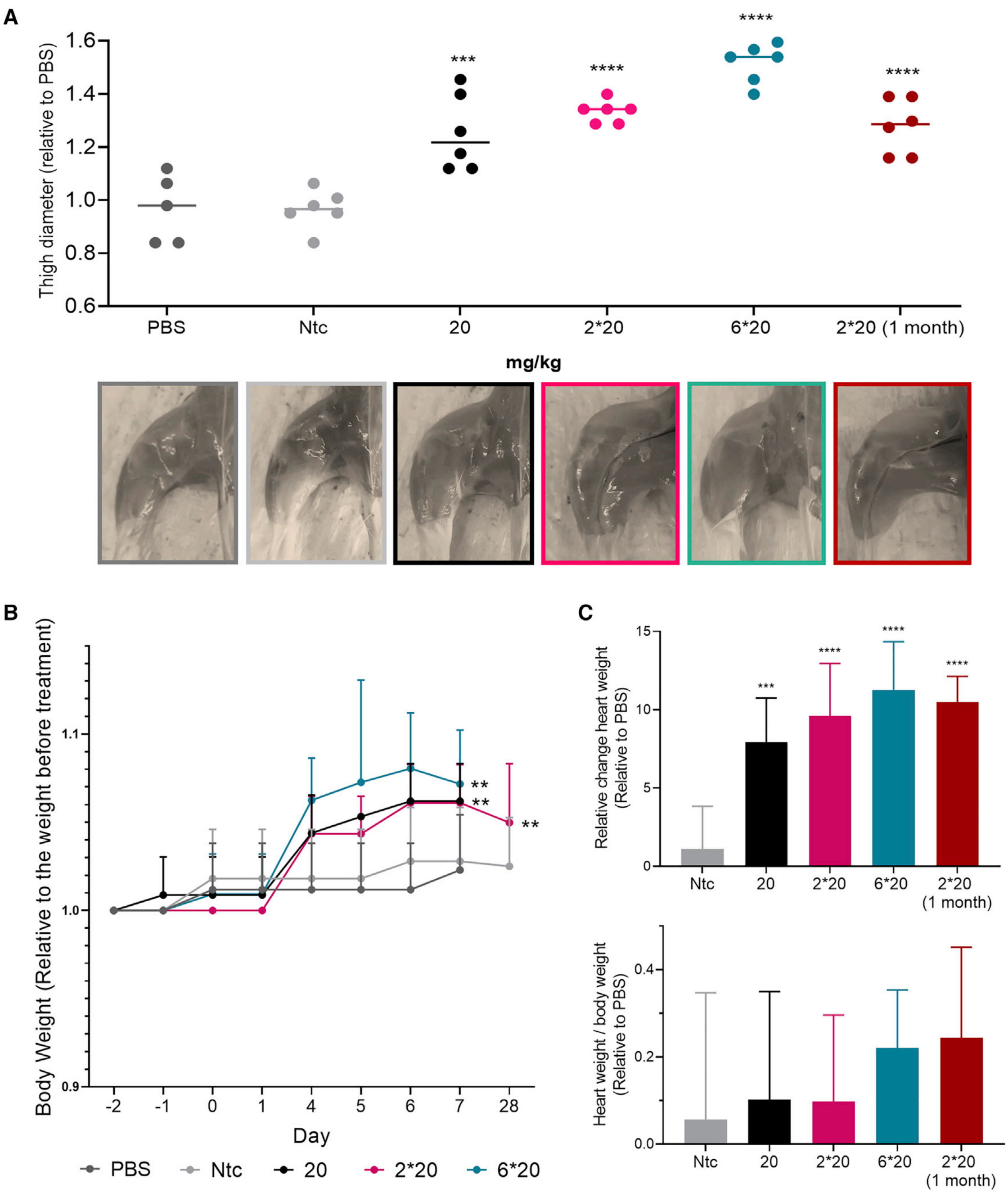

Figure 4. Mstn silencing in muscles leads to a significant increase in muscle growth

(A) Thigh diameter normalized to PBS and representative images of mouse hindlimbs after injection at various doses. (B) Body weight normalized to body weight at day −2. (C) Relative change in heart weight compared with PBS group and heart weight/body weight ratio normalized to the PBS group. s.c. injection of DCA-conjugated siRNA in mice sacrificed at 1 week and 1 month post-injection (n = 6 mice per group, mean ± SD; 20, 2 × 20, and 6 × 20 mg/kg). Data analysis: multiple comparisons = one-way ANOVA, Dunnett test and two-way ANOVA, Tukey test (****$p < 0.0001$, ***$p < 0.001$, **$p < 0.01$).

may result in a different toxicity profile. Therefore, cytokine levels have been evaluated in mice (n = 3) after either a single injection (20 mg/kg) or multiple injections (2 × 20 and 6 × 20 mg/kg) of DCA-conjugated siRNAs (Figure 6; Figure S6). For all the dose regimens tested, no significant cytokine elevation has been observed, except for the chemokine MCP-3 ($p < 0.001$) after six injections of 20 mg/kg. These results confirmed that DCA conjugate did not induce a significant increase in cytokine levels, demonstrating that

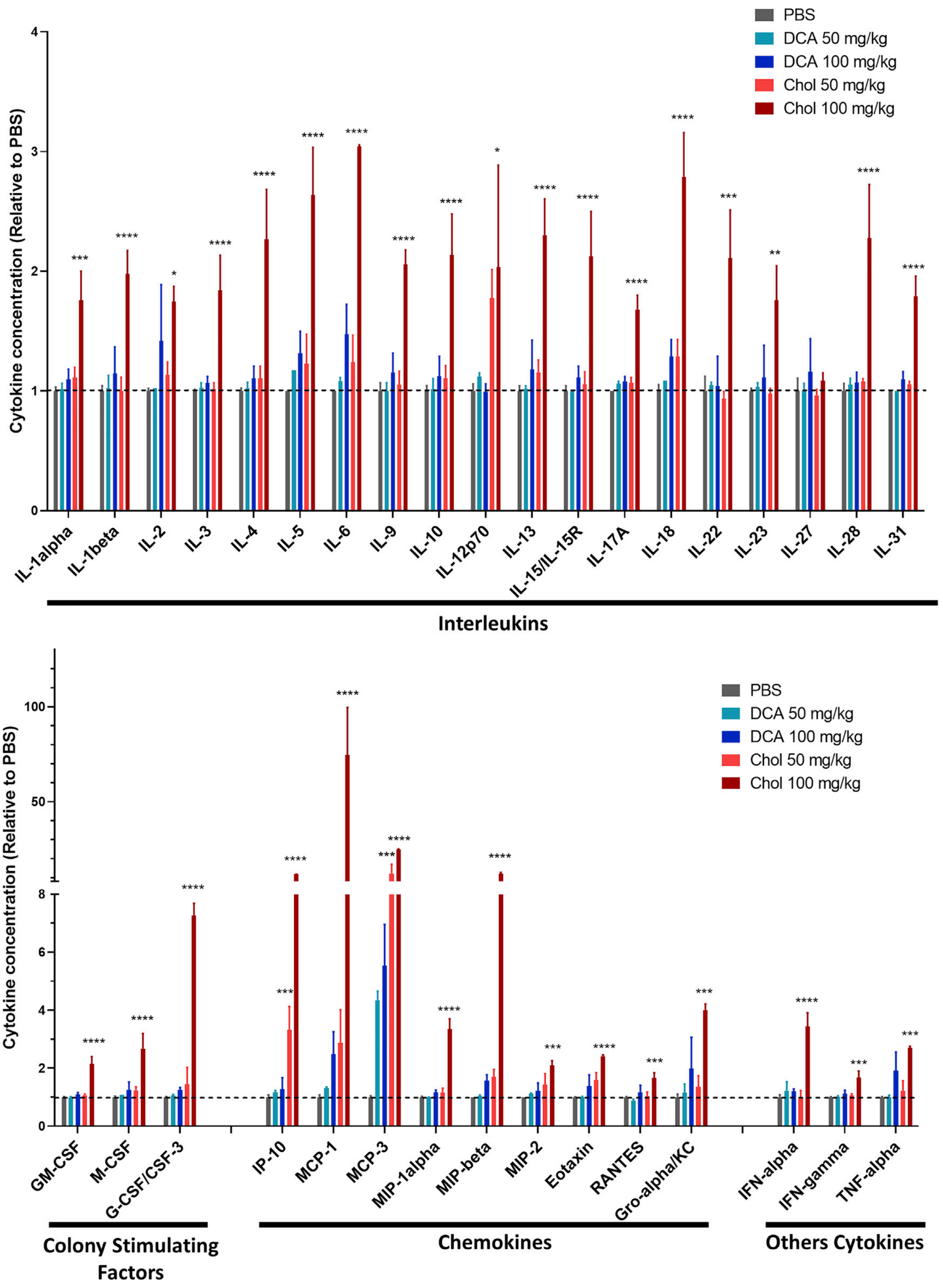

Figure 5. DCA conjugate shows a safe cytokine profile at high doses

Bar graph showing cytokine levels relative to PBS. 24 h after s.c. injections of DCA- and cholesterol-conjugated siRNAs at 50 and 100 mg/kg (n = 3 mice per group, mean ± SD). Data analysis: multiple comparisons = one-way ANOVA, Dunnett test (****$p < 0.0001$, ***$p < 0.001$, **$p < 0.01$, *$p < 0.1$).

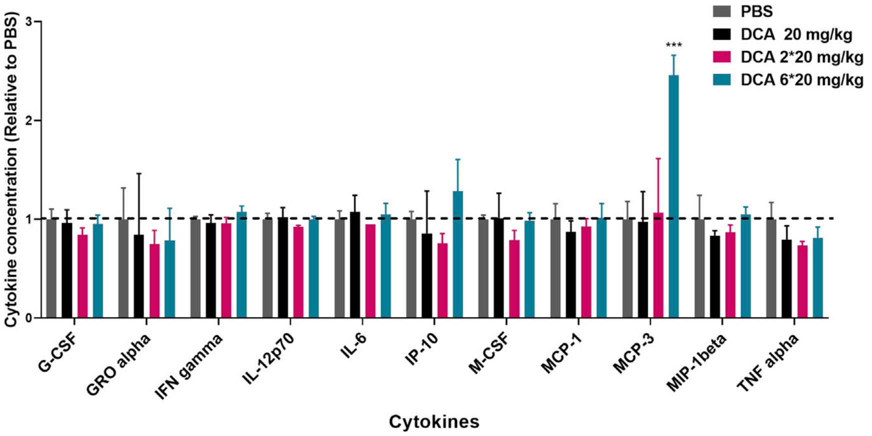

Figure 6. DCA conjugate shows a safe cytokine profile after multiple injections

Bar graph showing cytokine levels relative to PBS. 1 week after s.c. injections of DCA-conjugated siRNAs at 20, 2 × 20, and 6 × 20 mg/kg (n = 3 mice per group, mean ± SD). Data analysis: multiple comparisons = one-way ANOVA, Dunnett test (****$p < 0.0001$, ***$p < 0.001$, **$p < 0.01$, *$p < 0.1$).

multiple dosing of DCA compounds can be performed without concern of toxicity.

DISCUSSION

Although transformative for liver delivery, GalNAc conjugation does not allow significant oligonucleotide accumulation in tissues other than liver and kidneys.[19,57] Inefficient systemic delivery of oligonucleotides to skeletal and cardiac muscle remains a major roadblock in the development of sustainable drugs for muscle disorders.[26,58,59] Several ASOs are currently in pre-clinical and clinical development for the treatment of multiple muscle diseases;[60,61] however, their inefficient delivery to muscle requires the use of higher doses than that used for GalNAc compounds in liver,[62] resulting in dose-limiting toxicities.[56] We and others have shown that fatty acid conjugation to oligonucleotides supports broad tissue distribution and induces silencing in several extrahepatic tissues;[9,32–34,36–39,46] thus, it represents a viable approach for improving systemic delivery of compounds to muscles. In our previous reports,[32,33] we demonstrate that DCA is optimal for efficient delivery to, and sustainable gene silencing in, skeletal and cardiac muscles. Here, we designed chemically optimized DCA-conjugated siRNAs to silence *Mstn* and optimized a dosing regimen for muscle delivery. *Mstn* silencing by DCA-siRNAs led to muscle growth without causing toxicity, validating the therapeutic potential of this compound. Our findings provide a foundation for developing efficient therapeutic siRNA for the treatment of muscle disorders.

Previous reports clearly demonstrate the impact of conjugate structure on the extrahepatic tissue distribution and efficacy of siRNAs. The higher accumulation of DCA-siRNA in muscle compared with unconjugated (~7 fold) and other conjugated siRNAs (e.g., ~2.5-fold higher than cholesterol-siRNAs) (Figure S1)[32,33] can be explained by differences in binding affinity to serum lipoproteins, which dictate siRNA plasma circulation and tissue exposure.[33,34,37,41,45,46,63] Long, saturated fatty acids like DCA bind low-density lipoprotein (LDL), whereas unsaturated and short-chain saturated fatty acids primarily bind high-density lipoprotein (HDL).[34,37,46] In muscle, the higher siRNA accumulation correlates to productive gene silencing, but the use of a cleavable linker (d(TT)) can further increase siRNA activity in muscle and heart (and other tissues) without changing accumulation.[47] This is likely because cleavable linkers enhance endosomal escape of conjugated oligonucleotides in cells.[42,64,65] Fine-tuning the conjugate and linker chemistries of siRNA is crucial to optimizing functional delivery in extrahepatic tissues.

In the context of ASOs, the same optimization principles may not apply. Prakash et al.[37] found that high binding affinity to plasma albumin (not HDL/LDL) correlated to high ASO activity in muscles. Moreover, the highest protein binding affinity was observed with fatty acid chain lengths from 16 to 18 carbons, with degree of unsaturation having no influence. These differences in protein binding and fatty acid structure that impact efficacy may be because of the difference in phosphorothioate (PS) content, which has been shown to impact protein binding, in ASOs compared with siRNAs. ASOs are single-stranded fully phosphorothioated compounds, making these modifications a significant contributor to protein binding.[15,66,67] siRNAs, in contrast, are only partially phosphorothioated; thus, the relative contribution of PS modifications to protein binding (~9–10 continuous modifications enables protein association[68]) and tissue distribution[42] may be smaller than that of the conjugate structure. Interestingly, Prakash et al.[37] also did not observe changes in ASO activity with a cleavable d(TCA) linker, indicating that chemical structure differences between two classes of oligonucleotide (ASO versus siRNA) have a profound impact on PK/PD properties, and optimization principles may be different between ASOs and siRNAs.

In this study, we build on our previous siRNA findings by demonstrating that observed gene silencing in skeletal and cardiac muscles after systemic injection of DCA-conjugated siRNAs is not target specific. DCA-conjugated siRNAs induced robust and prolonged Mstn mRNA and protein silencing in muscles, leading to increased muscle growth. The observed silencing and change in phenotype were still maintained even 1 month after injection, demonstrating sustained efficacy. The accumulation in cardiac muscle was superior (~1.5 pmol/mg) relative to skeletal muscles (~0.4 pmol/mg), and 25% of siRNAs present at 1 week post-injection remained at 1 month (~0.38 pmol/mg), sufficient to maintain ~65% silencing. Why does heart accumulate more siRNA than skeletal muscles? Although both skeletal and cardiac muscles have generally similar endothelial structure, small changes in endothelial cell number and arrangement (which can be

affected by a disease state) might significantly impact accumulation and efficacy. Moreover, blood volumes and overall drug exposure are naturally higher in heart.

An interesting phenomenon observed in this current study is siRNA delivery saturation: above certain levels of siRNA accumulation in tissues and within certain periods of time, additional dosing of siRNA does not increase compound accumulation or efficacy in tissues. This saturation phenomenon, which has not previously been reported, presents differently in different tissues. In liver, an extra dose administrated within 12 h resulted in a 2-fold increase in accumulation, indicating that the initial dose of oligonucleotides (20 mg/kg) either did not saturate the liver or liver uptake mechanisms recovered over a 12-h period. Additional doses delivered within the next 48 h did not proportionally increase accumulation, suggesting two doses within 12 h might be optimal to deliver siRNAs. By contrast, only a single dose was needed to fully saturate the heart. It is clear that the maximum amount of oligonucleotide uptake is tissue specific: only ~1.8 pmol/mg was needed to reach saturation in heart, whereas ~27 pmol/mg did not fully saturate liver. Liver is a primary clearance tissue that relies on multiple mechanisms of internalization for blood/tissue exchange. These mechanisms of uptake in liver may not translate to equivalent levels of productive RNA-induced silencing complex (RISC) loading and silencing. In the heart, the extent of blood exchange is significantly less, but the uptake mechanisms may provide better functional access. Indeed, we and others have previously reported the disproportionally higher level of compound accumulation necessary to achieve productive silencing in primary clearance tissues compared with other tissues, such as muscle.[32]

In skeletal muscles, we observed a third saturation scenario. An extra dose of siRNA within 12 h had no impact on accumulation, suggesting the tissue was still saturated 12 h after injection. However, additional doses within the next 48 h resulted in a ~2.5-fold increase in accumulation. Collectively, these results advance our current knowledge of siRNA development by demonstrating that the dosing regimen of siRNAs also needs to be optimized to support optimal duration of effect in a specific tissue of interest.

When targeting *Mstn*, DCA-siRNA achieved silencing levels that supported profound changes in phenotype. The degree of silencing was proportional to the injected dose and to muscle volume growth. In addition, both the degree of muscle silencing and the degree of muscle increase were consistent over a period from 1 week to 1 month. These data demonstrate that specific dosing can be used to achieve and maintain different degrees of target modulation.

Accumulation is predictive of duration of effect.[11,13,52] We observed ~0.12 pmol/mg siRNAs in skeletal muscles after 1 month post-injection, which was still sufficient to maintain ~40% silencing. In heart tissue, we observed higher accumulation: at 1 month, there was still ~0.4 pmol/mg siRNAs in the tissue, which is likely to support silencing for more than 2 months in rodents with a single injection. Further studies will need to be performed to estimate the limits of the duration of effect. At this point, it is estimated that monthly dosing in mice would be sufficient to maintain activity. With siRNAs and ASOs, the potency and duration of effect seem to increase with translation from rodents to non-human primates to humans. For example, with inclisiran, a 3-week duration of effect in rodents translated into more than 6 months of efficacy in humans.[8,25] Thus, it is possible that the sustained modulation of Mstn expression in muscles and heart might show much longer duration of effect in humans, which has important implications for therapeutic applications.

In tissues such as muscles, tissue damage can be caused by the disease, leading to an increase of oligonucleotide accumulation in muscular tissues. Therefore, we expect that the DCA performance to deliver siRNA to muscles observed here in healthy mice will be better in disease models. With that said, it is always unknown how the disease state might impact siRNA accumulation, and thus the exact distribution and efficacy will need to be confirmed in disease models.

In addition, distribution and efficacy of hydrophobic-conjugated siRNAs (e.g., DCA-conjugated siRNAs) rely on serum protein composition, which may be heavily affected by the animal diet. In the future, it would be interesting to systematically explore how high-fat diet (increase in LDL content) versus starvation will impact distribution of this class of oligonucleotides, which, theoretically, can be substantial.

Although hydrophobic conjugation supports wide extrahepatic delivery[9,32] and, in the context of structurally optimized siRNAs, can induce functional relevant silencing,[42] toxicity concerns have limited their therapeutic translation. Indeed, cholesterol-conjugated siRNAs, the only conjugate reported to deliver siRNAs to muscles after systemic administration,[38] induce a substantial cytokine storm (up to 80-fold increases for certain markers) at high doses (~100 mg/kg). By contrast, DCA-siRNAs did not induce cytokines or any other observable adverse events at the same high dose (100 mg/kg), indicating superior safety. However, like other lipid-conjugated siRNAs, DCA-siRNAs predominantly accumulate in clearance tissues (~77% of the injected dose cumulatively). Thus, it is essential that DCA-mediated modulation of potential therapeutic targets in clearance tissues is not expected to cause any detrimental effects.[36] Alternatively, the technology may be optimal in contexts where the target genes are selectively expressed in muscle and heart, like Mstn[48] or DUX4.[69]

As of the publication of this article, only ASO-mediated splicing modulations have been approved by the FDA to treat muscle-related diseases, such as Duchenne muscular dystrophy (DMA) (eteplirsen and golodirsen) and spinal muscular atrophy (SMA) (nusinersen).[70] Promising data have been reported, showing an increase in dystrophin expression.[26,71] Although encouraging clinical results have been observed, the limited ASO delivery to muscles requires the use of high doses, which can lead to renal toxicity,[71] and thus limiting the use of ASO for the treatment of muscle-related diseases.[56] The DCA platform described in this manuscript allows efficient

oligonucleotide delivery in muscles after systemic administration, leading to significant target reduction in both cardiac and skeletal muscles (up to 80%) and enabling a change in phenotype without causing any toxicity even at high doses. Therefore, DCA conjugation provides a safe, sustainable, and robust alternative to treat muscle disorders. In summary, findings from our study demonstrate that DCA conjugated to structurally optimized siRNA scaffolds can support efficient, sustained, and safe delivery to muscle and heart after systemic administration. These findings establish a path toward using RNAi technology for functional genomic studies in muscle and heart and developing novel therapeutic paradigms for muscle disorders.

MATERIALS AND METHODS

General method for oligonucleotide synthesis

Oligonucleotides were synthesized on a Mermaid 12 synthesizer following standard protocols. In brief, conjugated sense strands were synthesized at 10-μmol scales on custom-synthesized lipid-functionalized controlled pore glass (CPG) supports[32,33,72] and were cleaved and deprotected using 40% aq. methylamine at 45°C for 1 h. Antisense strands were synthesized at 10-μmol scales on CPG functionalized with Unylinker (ChemGenes, Wilmington, MA, USA). They were first deprotected with a solution of bromotrimethylsilane/pyridine (3:2, v/v) in dichloromethane for the (E)-vinylphosphonate deprotection, then cleaved and deprotected with 40% aq. methylamine at 45°C for 1 h. Oligonucleotides were purified using an Agilent Prostar System (Agilent, Santa Clara, CA, USA) over a C18 column for lipid-conjugated sense strands and over an ion-exchange column for antisense strands. Purified oligonucleotides were desalted by size-exclusion chromatography and characterized by liquid chromatography-mass spectrometry (LC/MS) analysis on an Agilent 6530 accurate-mass quadrupole time-of-flight (Q-TOF) LC/MS (Agilent Technologies, Santa Clara, CA, USA).

Injection of lipid-conjugated siRNAs into mice

Animal experiments were performed in accordance with animal care ethics approval and guidelines of University of Massachusetts Medical School Institutional Animal Care and Use Committee (IACUC; protocol number A-2411). In all experiments, 7-week-old female FVB/NJ mice were used and were injected s.c. with either phosphate-buffered saline (PBS controls), non-targeting control siRNA (*Ntc*), or lipid-conjugated siRNA (n = 6 per group).

PNA hybridization assay

Quantification of antisense strands in tissues was performed using a PNA hybridization assay as described.[45,73] In brief, tissues (10 mg) were lysed in 200 μL MasterPure tissue lysis solution (EpiCentre) containing 0.2 mg/mL Proteinase K (Invitrogen). Sodium dodecyl sulfate (SDS) was precipitated from lysates by adding 20 μL 3 M potassium chloride and pelleted centrifugation at 5,000 × *g* for 15 min. Lipid-conjugated siRNAs in cleared supernatant were hybridized to a Cy3-labeled PNA probe fully complementary to the antisense strand (PNABio; Thousand Oaks, CA, USA). Samples were analyzed by high-performance liquid chromatography (HPLC; Agilent, Santa Clara, CA, USA) over a DNAPac PA100 anion-exchange column (Thermo Fisher Scientific). Cy3 fluorescence was monitored and peaks integrated. Final concentrations were ascertained using calibration curves.

In vivo mRNA silencing experiments

At 1 week post-injection or 1 month post-injection, tissues were collected and stored in RNAlater (Sigma) at 4°C overnight. mRNA was quantified using the QuantiGene 2.0 Assay (Affymetrix). Tissue punches were lysed in 300 μL Homogenizing Buffer (Affymetrix) containing 0.2 mg/mL Proteinase K (Invitrogen). Diluted lysates and probe sets (mouse *Htt*, mouse *Mstn*, or mouse *Hprt*) were added to the bDNA capture plate, and the signal was amplified and detected as described by Coles et al.[74] Luminescence was detected on a Tecan M1000 (Tecan, Morrisville, NC, USA).

In vivo protein silencing experiments

Blood was collected by terminal cardiac puncture, and serum was analyzed for MSTN protein using the GDF-8/Myostatin Quantikine ELISA kit (R&D Systems, Minneapolis, MN, USA). Serum samples were activated as described in the manufacturer's protocol, with the exception that the final activated serum sample had an additional 1:2 dilution in calibrator diluent before assaying.

Cytokine level measurement

Mice were injected s.c. with DCA- and Chol-conjugated siRNAs at both 50 and 100 mg/kg. At 24 h post-injection, blood was collected by terminal cardiac puncture, and serum was analyzed for cytokine concentration measurement using Customized Luminex Assay (R&D Systems, Minneapolis, MN, USA). Serum samples were analyzed as described in the manufacturer's protocol.

Statistical analysis

Data were analyzed using GraphPad Prism 7.01 software (GraphPad Software, San Diego, CA, USA). For each independent experiment in mice, the level of silencing was normalized to the mean of the PBS control group. Data were analyzed using non-parametric one-way ANOVA with Dunnett test for multiple comparisons, with significance calculated relative to PBS controls and t test for comparison of two groups.

SUPPLEMENTAL INFORMATION

Supplemental Information can be found online at https://doi.org/10.1016/j.ymthe.2020.12.023.

ACKNOWLEDGMENTS

We thank all Khvorova lab members for insightful discussions and support, and Emily Haberlin for helping with the manuscript writing and editing. This work was supported by National Institutes of Health grants R01 HD086111, S10 OD020012, and R35 GM131839.

AUTHOR CONTRIBUTIONS

A.B. and A.K. conceived the project, designed the study, and wrote the manuscript. A.B. synthesized most of the compounds and performed most of the experiments and data analysis. J.C. helped with mouse

handling. N.M. contributed to compound synthesis. V.H. and M.M helped with cytotoxicity studies.

DECLARATION OF INTERESTS

A.K. owns stock of RXi Pharmaceuticals and Advirna. The other authors declare no competing interests.

REFERENCES

1. Zhou, J., Shum, K.-T., Burnett, J.C., and Rossi, J.J. (2013). Nanoparticle-based delivery of RNAi therapeutics: progress and challenges. Pharmaceuticals (Basel) *6*, 85–107.
2. Nair, J.K., Willoughby, J.L.S., Chan, A., Charisse, K., Alam, M.R., Wang, Q., Hoekstra, M., Kandasamy, P., Kel'in, A.V., Milstein, S., et al. (2014). Multivalent N-acetylgalactosamine-conjugated siRNA localizes in hepatocytes and elicits robust RNAi-mediated gene silencing. J. Am. Chem. Soc. *136*, 16958–16961.
3. Zimmermann, T.S., Karsten, V., Chan, A., Chiesa, J., Boyce, M., Bettencourt, B.R., Hutabarat, R., Nochur, S., Vaishnaw, A., and Gollob, J. (2017). Clinical proof of concept for a novel hepatocyte-targeting GalNAc-siRNA conjugate. Mol. Ther. *25*, 71–78.
4. Jackson, A.L., Burchard, J., Leake, D., Reynolds, A., Schelter, J., Guo, J., Johnson, J.M., Lim, L., Karpilow, J., Nichols, K., et al. (2006). Position-specific chemical modification of siRNAs reduces "off-target" transcript silencing. RNA *12*, 1197–1205.
5. Akinc, A., Maier, M.A., Manoharan, M., Fitzgerald, K., Jayaraman, M., Barros, S., Ansell, S., Du, X., Hope, M.J., Madden, T.D., et al. (2019). The Onpattro story and the clinical translation of nanomedicines containing nucleic acid-based drugs. Nat. Nanotechnol. *14*, 1084–1087.
6. Setten, R.L., Rossi, J.J., and Han, S.P. (2019). The current state and future directions of RNAi-based therapeutics. Nat. Rev. Drug Discov. *18*, 421–446.
7. Fitzgerald, K., White, S., Borodovsky, A., Bettencourt, B.R., Strahs, A., Clausen, V., Wijngaard, P., Horton, J.D., Taubel, J., Brooks, A., et al. (2017). A Highly Durable RNAi Therapeutic Inhibitor of PCSK9. N. Engl. J. Med. *376*, 41–51.
8. Ray, K.K., Wright, R.S., Kallend, D., Koenig, W., Leiter, L.A., Raal, F.J., Bisch, J.A., Richardson, T., Jaros, M., Wijngaard, P.L.J., and Kastelein, J.J.P.; ORION-10 and ORION-11 Investigators (2020). Two Phase 3 Trials of Inclisiran in Patients with Elevated LDL Cholesterol. N. Engl. J. Med. *382*, 1507–1519.
9. Osborn, M.F., and Khvorova, A. (2018). Improving siRNA delivery In vivo through Lipid conjugation. Nucleic Acid Ther. *28*, 128–136.
10. Soutschek, J., Akinc, A., Bramlage, B., Charisse, K., Constien, R., Donoghue, M., Elbashir, S., Geick, A., Hadwiger, P., Harborth, J., et al. (2004). Therapeutic silencing of an endogenous gene by systemic administration of modified siRNAs. Nature *432*, 173–178.
11. Foster, D.J., Brown, C.R., Shaikh, S., Trapp, C., Schlegel, M.K., Qian, K., Sehgal, A., Rajeev, K.G., Jadhav, V., Manoharan, M., et al. (2018). Advanced siRNA designs further improve in vivo performance of GalNAc-siRNA conjugates. Mol. Ther. *26*, 708–717.
12. Allerson, C.R., Sioufi, N., Jarres, R., Prakash, T.P., Naik, N., Berdeja, A., Wanders, L., Griffey, R.H., Swayze, E.E., and Bhat, B. (2005). Fully 2′-modified oligonucleotide duplexes with improved in vitro potency and stability compared to unmodified small interfering RNA. J. Med. Chem. *48*, 901–904.
13. Nair, J.K., Attarwala, H., Sehgal, A., Wang, Q., Aluri, K., Zhang, X., Gao, M., Liu, J., Indrakanti, R., Schofield, S., et al. (2017). Impact of enhanced metabolic stability on pharmacokinetics and pharmacodynamics of GalNAc-siRNA conjugates. Nucleic Acids Res. *45*, 10969–10977.
14. Nallagatla, S.R., and Bevilacqua, P.C. (2008). Nucleoside modifications modulate activation of the protein kinase PKR in an RNA structure-specific manner. RNA *14*, 1201–1213.
15. Geary, R.S., Norris, D., Yu, R., and Bennett, C.F. (2015). Pharmacokinetics, biodistribution and cell uptake of antisense oligonucleotides. Adv. Drug Deliv. Rev. *87*, 46–51.
16. Eckstein, F. (2002). Developments in RNA chemistry, a personal view. Biochimie *84*, 841–848.
17. Haraszti, R.A., Roux, L., Coles, A.H., Turanov, A.A., Alterman, J.F., Echeverria, D., Godinho, B.M.D.C., Aronin, N., and Khvorova, A. (2017). 5′-Vinylphosphonate improves tissue accumulation and efficacy of conjugated siRNAs in vivo. Nucleic Acids Res. *45*, 7581–7592.
18. Parmar, R., Willoughby, J.L.S., Liu, J., Foster, D.J., Brigham, B., Theile, C.S., Charisse, K., Akinc, A., Guidry, E., Pei, Y., et al. (2016). 5′-(E)-Vinylphosphonate: a stable phosphate mimic can improve the RNAi activity of siRNA–GalNAc conjugates. ChemBioChem *17*, 985–989.
19. Hassler, M.R., Turanov, A.A., Alterman, J.F., Haraszti, R.A., Coles, A.H., Osborn, M.F., Echeverria, D., Nikan, M., Salomon, W.E., Roux, L., et al. (2018). Comparison of partially and fully chemically-modified siRNA in conjugate-mediated delivery in vivo. Nucleic Acids Res. *46*, 2185–2196.
20. Huang, Y. (2017). Preclinical and clinical advances of GalNAc-decorated nucleic acid therapeutics. Mol. Ther. Nucleic Acids *6*, 116–132.
21. Rajeev, K.G., Nair, J.K., Jayaraman, M., Charisse, K., Taneja, N., O'Shea, J., Willoughby, J.L., Yucius, K., Nguyen, T., Shulga-Morskaya, S., et al. (2015). Hepatocyte-specific delivery of siRNAs conjugated to novel non-nucleosidic trivalent N-acetylgalactosamine elicits robust gene silencing in vivo. ChemBioChem *16*, 903–908.
22. Matsuda, S., Keiser, K., Nair, J.K., Charisse, K., Manoharan, R.M., Kretschmer, P., Peng, C.G., V Kel'in, A., Kandasamy, P., Willoughby, J.L., et al. (2015). siRNA conjugates carrying sequentially assembled trivalent N-acetylgalactosamine linked through nucleosides elicit robust gene silencing in vivo in hepatocytes. ACS Chem. Biol. *10*, 1181–1187.
23. Prakash, T.P., Graham, M.J., Yu, J., Carty, R., Low, A., Chappell, A., Schmidt, K., Zhao, C., Aghajan, M., Murray, H.F., et al. (2014). Targeted delivery of antisense oligonucleotides to hepatocytes using triantennary N-acetyl galactosamine improves potency 10-fold in mice. Nucleic Acids Res. *42*, 8796–8807.
24. Tanowitz, M., Hettrick, L., Revenko, A., Kinberger, G.A., Prakash, T.P., and Seth, P.P. (2017). Asialoglycoprotein receptor 1 mediates productive uptake of N-acetylgalactosamine-conjugated and unconjugated phosphorothioate antisense oligonucleotides into liver hepatocytes. Nucleic Acids Res. *45*, 12388–12400.
25. Raal, F.J., Kallend, D., Ray, K.K., Turner, T., Koenig, W., Wright, R.S., Wijngaard, P.L.J., Curcio, D., Jaros, M.J., Leiter, L.A., and Kastelein, J.J.P.; ORION-9 Investigators (2020). Inclisiran for the Treatment of Heterozygous Familial Hypercholesterolemia. N. Engl. J. Med. *382*, 1520–1530.
26. Aartsma-Rus, A., and Krieg, A.M. (2017). FDA Approves Eteplirsen for Duchenne Muscular Dystrophy: The Next Chapter in the Eteplirsen Saga. Nucleic Acid Ther. *27*, 1–3.
27. Wood, M.J.A., Talbot, K., and Bowerman, M. (2017). Spinal muscular atrophy: antisense oligonucleotide therapy opens the door to an integrated therapeutic landscape. Hum. Mol. Genet. *26* (R2), R151–R159.
28. Gebski, B.L., Mann, C.J., Fletcher, S., and Wilton, S.D. (2003). Morpholino antisense oligonucleotide induced dystrophin exon 23 skipping in mdx mouse muscle. Hum. Mol. Genet. *12*, 1801–1811.
29. Hagstrom, J.E., Hegge, J., Zhang, G., Noble, M., Budker, V., Lewis, D.L., Herweijer, H., and Wolff, J.A. (2004). A facile nonviral method for delivering genes and siRNAs to skeletal muscle of mammalian limbs. Mol. Ther. *10*, 386–398.
30. Kawakami, E., Kawai, N., Kinouchi, N., Mori, H., Ohsawa, Y., Ishimaru, N., Sunada, Y., Noji, S., and Tanaka, E. (2013). Local applications of myostatin-siRNA with atelocollagen increase skeletal muscle mass and recovery of muscle function. PLoS ONE *8*, e64719.
31. Dowdy, S.F. (2017). Overcoming cellular barriers for RNA therapeutics. Nat. Biotechnol. *35*, 222–229.
32. Biscans, A., Coles, A., Haraszti, R., Echeverria, D., Hassler, M., Osborn, M., and Khvorova, A. (2019). Diverse lipid conjugates for functional extra-hepatic siRNA delivery in vivo. Nucleic Acids Res. *47*, 1082–1096.
33. Biscans, A., Coles, A., Echeverria, D., and Khvorova, A. (2019). The valency of fatty acid conjugates impacts siRNA pharmacokinetics, distribution, and efficacy in vivo. J. Control. Release *302*, 116–125.
34. Osborn, M.F., Coles, A.H., Biscans, A., Haraszti, R.A., Roux, L., Davis, S., Ly, S., Echeverria, D., Hassler, M.R., Godinho, B.M.D.C., et al. (2019). Hydrophobicity

drives the systemic distribution of lipid-conjugated siRNAs via lipid transport pathways. Nucleic Acids Res. *47*, 1070–1081.

35. Yuan, H., Lanting, L., Xu, Z.-G., Li, S.-L., Swiderski, P., Putta, S., Jonnalagadda, M., Kato, M., and Natarajan, R. (2008). Effects of cholesterol-tagged small interfering RNAs targeting 12/15-lipoxygenase on parameters of diabetic nephropathy in a mouse model of type 1 diabetes. Am. J. Physiol. Renal Physiol. *295*, F605–F617.

36. Turanov, A.A., Lo, A., Hassler, M.R., Makris, A., Ashar-Patel, A., Alterman, J.F., Coles, A.H., Haraszti, R.A., Roux, L., Godinho, B.M.D.C., et al. (2018). RNAi modulation of placental sFLT1 for the treatment of preeclampsia. Nat. Biotechnol. *36*, 1164–1173.

37. Prakash, T.P., Mullick, A.E., Lee, R.G., Yu, J., Yeh, S.T., Low, A., Chappell, A.E., Østergaard, M.E., Murray, S., Gaus, H.J., et al. (2019). Fatty acid conjugation enhances potency of antisense oligonucleotides in muscle. Nucleic Acids Res. *47*, 6029–6044.

38. Khan, T., Weber, H., DiMuzio, J., Matter, A., Dogdas, B., Shah, T., Thankappan, A., Disa, J., Jadhav, V., Lubbers, L., et al. (2016). Silencing myostatin using cholesterol-conjugated siRNAs induces muscle growth. Mol. Ther. Nucleic Acids *5*, e342.

39. Østergaard, M.E., Jackson, M., Low, A., E Chappell, A., G Lee, R., Peralta, R.Q., Yu, J., Kinberger, G.A., Dan, A., Carty, R., et al. (2019). Conjugation of hydrophobic moieties enhances potency of antisense oligonucleotides in the muscle of rodents and non-human primates. Nucleic Acids Res. *47*, 6045–6058.

40. Van der Vusse, G.J., Glatz, J.F., Van Nieuwenhoven, F.A., Reneman, R.S., and Bassingthwaighte, J.B. (1998). Transport of long-chain fatty acids across the muscular endothelium. Adv. Exp. Med. Biol. *441*, 181–191.

41. Chappell, A.E., Gaus, H.J., Berdeja, A., Gupta, R., Jo, M., Prakash, T.P., Oestergaard, M., Swayze, E.E., and Seth, P.P. (2020). Mechanisms of palmitic acid-conjugated antisense oligonucleotide distribution in mice. Nucleic Acids Res. *48*, 4382–4395.

42. Biscans, A., Caiazzi, J., Davis, S., McHugh, N., Sousa, J., and Khvorova, A. (2020). The chemical structure and phosphorothioate content of hydrophobically modified siRNAs impact extrahepatic distribution and efficacy. Nucleic Acids Res. *48*, 7665–7680.

43. Eckstein, F. (2014). Phosphorothioates, essential components of therapeutic oligonucleotides. Nucleic Acid Ther. *24*, 374–387.

44. Gaus, H.J., Gupta, R., Chappell, A.E., Østergaard, M.E., Swayze, E.E., and Seth, P.P. (2019). Characterization of the interactions of chemically-modified therapeutic nucleic acids with plasma proteins using a fluorescence polarization assay. Nucleic Acids Res. *47*, 1110–1122.

45. Godinho, B.M.D.C., Gilbert, J.W., Haraszti, R.A., Coles, A.H., Biscans, A., Roux, L., Nikan, M., Echeverria, D., Hassler, M., and Khvorova, A. (2017). Pharmacokinetic profiling of conjugated therapeutic oligonucleotides: a high-throughput method based upon serial blood microsampling coupled to Peptide Nucleic Acid hybridization assay. Nucleic Acid Ther. *27*, 323–334.

46. Wolfrum, C., Shi, S., Jayaprakash, K.N., Jayaraman, M., Wang, G., Pandey, R.K., Rajeev, K.G., Nakayama, T., Charrise, K., Ndungo, E.M., et al. (2007). Mechanisms and optimization of in vivo delivery of lipophilic siRNAs. Nat. Biotechnol. *25*, 1149–1157.

47. Wickstrom, E. (1986). Oligodeoxynucleotide stability in subcellular extracts and culture media. J. Biochem. Biophys. Methods *13*, 97–102.

48. Tsuchida, K. (2008). Targeting myostatin for therapies against muscle-wasting disorders. Curr. Opin. Drug Discov. Devel. *11*, 487–494.

49. Rodgers, B.D., Interlichia, J.P., Garikipati, D.K., Mamidi, R., Chandra, M., Nelson, O.L., Murry, C.E., and Santana, L.F. (2009). Myostatin represses physiological hypertrophy of the heart and excitation-contraction coupling. J. Physiol. *587*, 4873–4886.

50. Kinouchi, N., Ohsawa, Y., Ishimaru, N., Ohuchi, H., Sunada, Y., Hayashi, Y., Tanimoto, Y., Moriyama, K., and Noji, S. (2008). Atelocollagen-mediated local and systemic applications of myostatin-targeting siRNA increase skeletal muscle mass. Gene Ther. *15*, 1126–1130.

51. Liu, C.-M., Yang, Z., Liu, C.-W., Wang, R., Tien, P., Dale, R., and Sun, L.Q. (2008). Myostatin antisense RNA-mediated muscle growth in normal and cancer cachexia mice. Gene Ther. *15*, 155–160.

52. Alterman, J.F., Godinho, B.M.D.C., Hassler, M.R., Ferguson, C.M., Echeverria, D., Sapp, E., Haraszti, R.A., Coles, A.H., Conroy, F., Miller, R., et al. (2019). A divalent siRNA chemical scaffold for potent and sustained modulation of gene expression throughout the central nervous system. Nat. Biotechnol. *37*, 884–894.

53. Reed, D.R., Bachmanov, A.A., and Tordoff, M.G. (2007). Forty mouse strain survey of body composition. Physiol. Behav. *91*, 593–600.

54. Wanke, R., Milz, S., Rieger, N., Ogiolda, L., Renner-Müller, I., Brem, G., Hermanns, W., and Wolf, E. (1999). Overgrowth of skin in growth hormone transgenic mice depends on the presence of male gonads. J. Invest. Dermatol. *113*, 967–971.

55. Taniguchi, T., Miyauchi, E., Nakamura, S., Hirai, M., Suzue, K., Imai, T., Nomura, T., Handa, T., Okada, H., Shimokawa, C., et al. (2015). Plasmodium berghei ANKA causes intestinal malaria associated with dysbiosis. Sci. Rep. *5*, 15699.

56. Goemans, N.M., Tulinius, M., van den Hauwe, M., Kroksmark, A.-K., Buyse, G., Wilson, R.J., van Deutekom, J.C., de Kimpe, S.J., Lourbakos, A., and Campion, G. (2016). Long-Term Efficacy, Safety, and Pharmacokinetics of Drisapersen in Duchenne Muscular Dystrophy: Results from an Open-Label Extension Study. PLoS ONE *11*, e0161955.

57. Shemesh, C.S., Yu, R.Z., Gaus, H.J., Greenlee, S., Post, N., Schmidt, K., Migawa, M.T., Seth, P.P., Zanardi, T.A., Prakash, T.P., et al. (2016). Elucidation of the Biotransformation Pathways of a Galnac3-conjugated Antisense Oligonucleotide in Rats and Monkeys. Mol. Ther. Nucleic Acids *5*, e319.

58. Spitali, P., and Aartsma-Rus, A. (2012). Splice modulating therapies for human disease. Cell *148*, 1085–1088.

59. Thornton, C.A., Wang, E., and Carrell, E.M. (2017). Myotonic dystrophy: approach to therapy. Curr. Opin. Genet. Dev. *44*, 135–140.

60. Tasfaout, H., Buono, S., Guo, S., Kretz, C., Messaddeq, N., Booten, S., Greenlee, S., Monia, B.P., Cowling, B.S., and Laporte, J. (2017). Antisense oligonucleotide-mediated Dnm2 knockdown prevents and reverts myotubular myopathy in mice. Nat. Commun. *8*, 15661.

61. Lieberman, A.P., Yu, Z., Murray, S., Peralta, R., Low, A., Guo, S., Yu, X.X., Cortes, C.J., Bennett, C.F., Monia, B.P., et al. (2014). Peripheral androgen receptor gene suppression rescues disease in mouse models of spinal and bulbar muscular atrophy. Cell Rep. *7*, 774–784.

62. Murray, S., Ittig, D., Koller, E., Berdeja, A., Chappell, A., Prakash, T.P., Norrbom, M., Swayze, E.E., Leumann, C.J., and Seth, P.P. (2012). TricycloDNA-modified oligo-2′-deoxyribonucleotides reduce scavenger receptor B1 mRNA in hepatic and extra-hepatic tissues–a comparative study of oligonucleotide length, design and chemistry. Nucleic Acids Res. *40*, 6135–6143.

63. Sarett, S.M., Werfel, T.A., Lee, L., Jackson, M.A., Kilchrist, K.V., Brantley-Sieders, D., and Duvall, C.L. (2017). Lipophilic siRNA targets albumin in situ and promotes bioavailability, tumor penetration, and carrier-free gene silencing. Proc. Natl. Acad. Sci. USA *114*, E6490–E6497.

64. Wada, S., Yasuhara, H., Wada, F., Sawamura, M., Waki, R., Yamamoto, T., Harada-Shiba, M., and Obika, S. (2016). Evaluation of the effects of chemically different linkers on hepatic accumulations, cell tropism and gene silencing ability of cholesterol-conjugated antisense oligonucleotides. J. Control. Release *226*, 57–65.

65. Zlatev, I., Castoreno, A., Brown, C.R., Qin, J., Waldron, S., Schlegel, M.K., Degaonkar, R., Shulga-Morskaya, S., Xu, H., Gupta, S., et al. (2018). Reversal of siRNA-mediated gene silencing in vivo. Nat. Biotechnol. *36*, 509–511.

66. Geary, R.S. (2009). Antisense oligonucleotide pharmacokinetics and metabolism. Expert Opin. Drug Metab. Toxicol. *5*, 381–391.

67. Oberbauer, R., Schreiner, G.F., and Meyer, T.W. (1995). Renal uptake of an 18-mer phosphorothioate oligonucleotide. Kidney Int. *48*, 1226–1232.

68. Brown, D.A., Kang, S.H., Gryaznov, S.M., DeDionisio, L., Heidenreich, O., Sullivan, S., Xu, X., and Nerenberg, M.I. (1994). Effect of phosphorothioate modification of oligodeoxynucleotides on specific protein binding. J. Biol. Chem. *269*, 26801–26805.

69. Ansseau, E., Vanderplanck, C., Wauters, A., Harper, S.Q., Coppée, F., and Belayew, A. (2017). Antisense oligonucleotides used to target the DUX4 mRNA as therapeutic approaches in FaciosScapuloHumeral Muscular Dystrophy (FSHD). Genes (Basel) *8*, 93.

70. Corey, D.R. (2017). Nusinersen, an antisense oligonucleotide drug for spinal muscular atrophy. Nat. Neurosci. *20*, 497–499.

71. Aartsma-Rus, A., and Corey, D.R. (2020). The 10th Oligonucleotide Therapy Approved: Golodirsen for Duchenne Muscular Dystrophy. Nucleic Acid Ther. *30*, 67–70.

72. Nikan, M., Osborn, M.F., Coles, A.H., Godinho, B.M., Hall, L.M., Haraszti, R.A., Hassler, M.R., Echeverria, D., Aronin, N., and Khvorova, A. (2016). Docosahexaenoic acid conjugation enhances distribution and safety of siRNA upon local administration in mouse brain. Mol. Ther. Nucleic Acids *5*, e344.

73. Roehl, I., Schuster, M., and Seiffert, S. (2011). Oligonucleotide detection method. US Patent US20110201006A1, filed October 6, 2009 and granted July 14, 2020.

74. Coles, A.H., Osborn, M.F., Alterman, J.F., Turanov, A.A., Godinho, B.M., Kennington, L., Chase, K., Aronin, N., and Khvorova, A. (2016). A high-throughput method for direct detection of therapeutic oligonucleotide-induced gene silencing in vivo. Nucleic Acid Ther. *26*, 86–92.

YMTHE, Volume *29*

Supplemental Information

Docosanoic acid conjugation to siRNA enables functional and safe delivery to skeletal and cardiac muscles

Annabelle Biscans, Jillian Caiazzi, Nicholas McHugh, Vignesh Hariharan, Manish Muhuri, and Anastasia Khvorova

A.

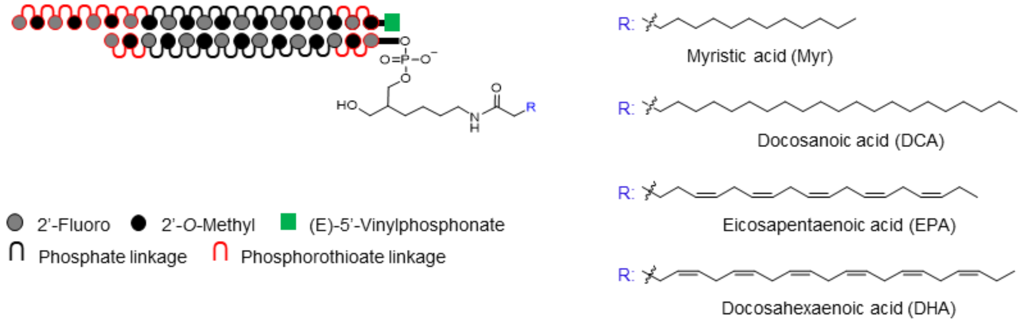

B.

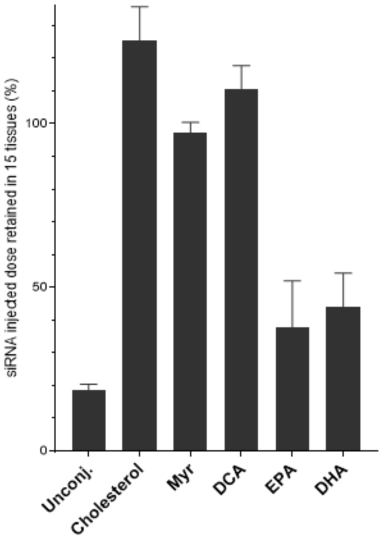

C.

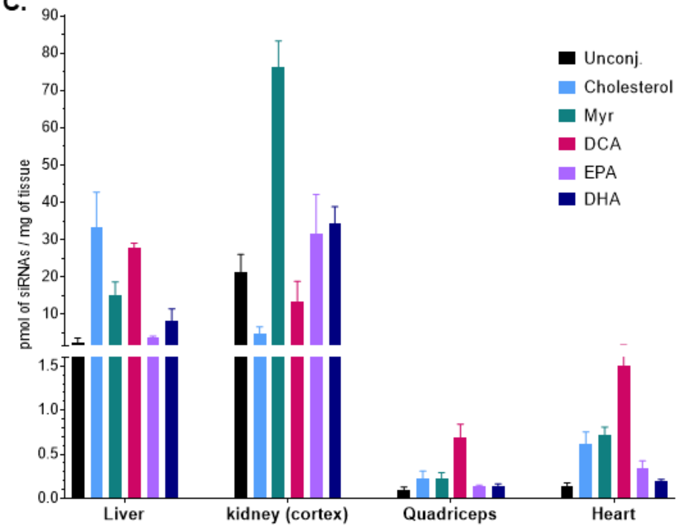

D.

Tissue	Conjugate	% injected dose	Tissue	Conjugate	% injected dose	Tissue	Conjugate	% injected dose	Tissue	Conjugate	% injected dose
Liver	Unconj.	6 ± 3	Kidneys	Unconj.	9 ± 2	Total skeletal muscles	Unconj.	0.6 ± 0.2	Heart	Unconj.	0.03 ± 0.007
	Cholesterol	80 ± 13		Cholesterol	2 ± 1		Cholesterol	1.4 ± 0.5		Cholesterol	0.15 ± 0.020
	Myr	39 ± 8		Myr	29 ± 3		Myr	1.4 ± 0.4		Myr	0.17 ± 0.020
	DCA	71 ± 3		DCA	6 ± 1		DCA	4.3 ± 0.9		DCA	0.40 ± 0.100
	EPA	10 ± 1		EPA	13 ± 3		EPA	1.3 ± 0.8		EPA	0.08 ± 0.020
	DHA	21 ± 8		DHA	13 ± 2		DHA	0.9 ± 0.2		DHA	0.05 ± 0.005

E.

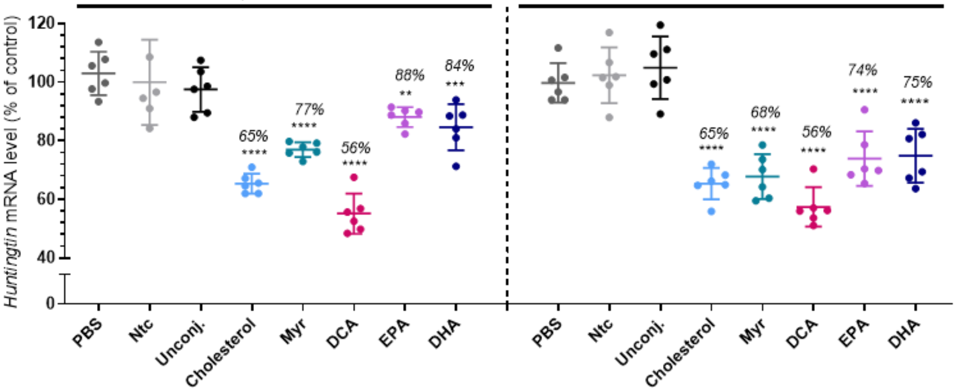

Fig. S1: Previously published data showing DCA conjugate allows quantitative retention in tissues, and significant accumulation and silencing in muscles after a single SC injection. (A.) Schematic representation of fatty acid conjugated siRNAs used to evaluate conjugate impact on muscle delivery (B.) Bar graph showing percent of injected dose retained in tissues at 48h post-injection (average of n = 3 ± SD) (C.) Bar graph showing siRNA quantification in liver, kidneys (cortex), quadriceps, and heart, measured by PNA hybridization assay (48h post-injection, 20 mg/kg, average of n = 3 ± SD) (D.) Table summarizing percent of injected dose retained in liver, kidneys, heart, and muscles at 48h post-injection (average of n= 3 ± SD) (E.) Percent of silencing in quadriceps and heart after SC injection of conjugated siRNA targeting *Huntingtin* (*Htt*) mRNA at 1-week (n=6 mice per group, 20 mg/kg). mRNA levels were measured using QuantiGene® (Affymetrix), normalized to a housekeeping gene, *Hprt* (Hypoxanthine-guanine phosphoribosyl transferase), and presented as percent of PBS (Phosphate buffered saline) control (mean ± SD). Data analysis: Multiple comparisons = One-way ANOVA, Dunnett test (****P<0.0001, ***P<0.001, **P<0.01). ***Data presented in this figure have been reformatted from previous publications (32,33).***

Tissue	Unconj. siRNA	Myr siRNA	DCA siRNA	EPA siRNA	DHA siRNA	Cholesterol siRNA
Liver	2.4 ± 1.3	15.3 ± 3.4	27.9 ± 1.2	3.9 ± 0.3	8.2 ± 3.3	33.3 ± 9.5
kidney (cortex)	21.3 ± 4.7	76.3 ± 7.0	13.5 ± 5.3	31.5 ± 10.6	34.3 ± 4.5	4.7 ± 1.9
Quadriceps	0.1 ± 0.0	0.2 ± 0.1	0.7 ± 0.1	0.1 ± 0.0	0.1 ± 0.0	0.2 ± 0.1
Heart	0.1 ± 0.0	0.7 ± 0.1	1.5 ± 0.3	0.3 ± 0.1	0.2 ± 0.0	0.6 ± 0.1

Fig. S2: DCA conjugate allows significant accumulation in both skeletal and cardiac tissues

Table showing siRNA quantification (pmol of siRNA / mg of tissue) in liver, kidneys (cortex), quadriceps, and heart, measured by PNA hybridization assay (48h post-injection, 20 mg/kg, average of n = 3 ± SD)

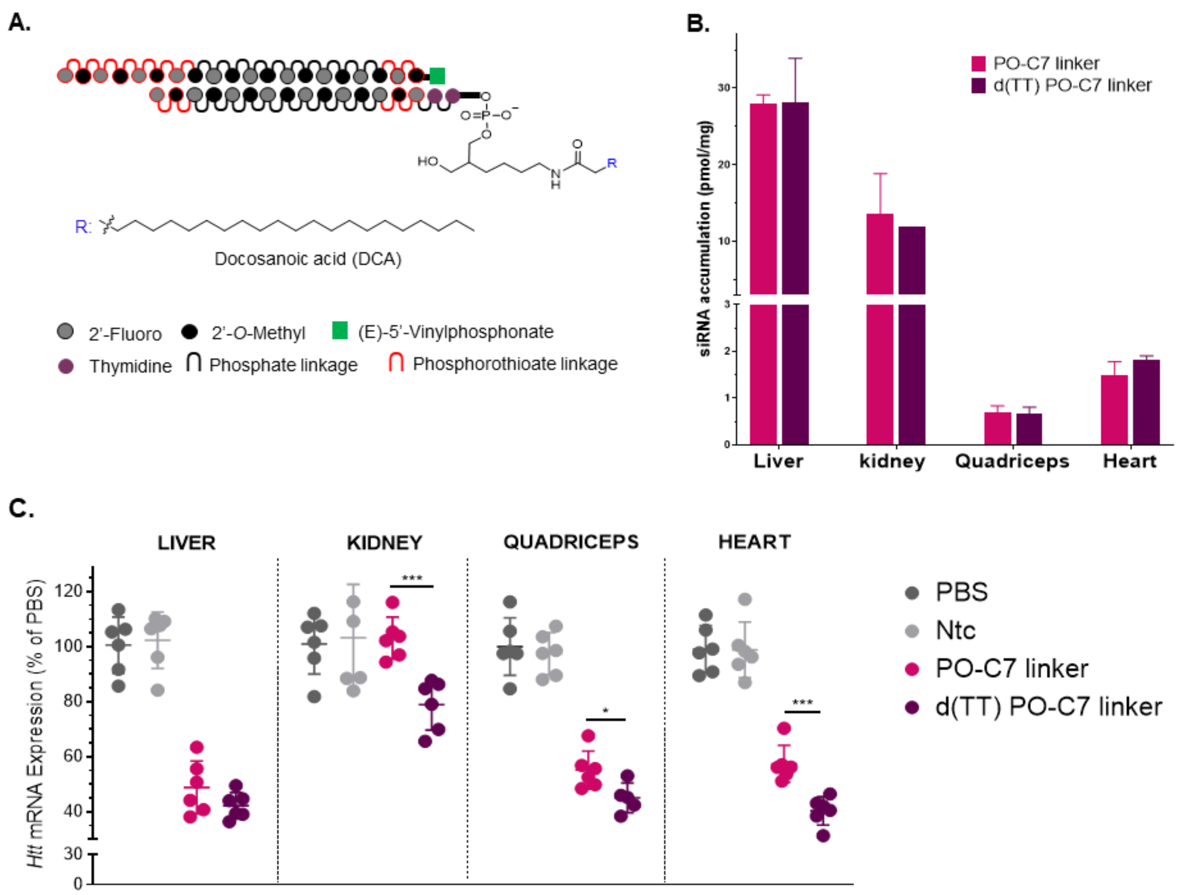

Fig. S3: Previously published data showing d(TT) PO-C7 linker enhanced DCA-conjugated siRNA silencing activity in muscles without affecting tissue accumulation. (A.) Schematic representation of DCA-conjugated siRNAs with d(TT) PO-C7 linker (B.) Bar graph showing siRNA quantification in liver, kidneys (cortex), quadriceps, and heart, measured by PNA hybridization assay (48h post-injection, 20 mg/kg, average of n = 3 ± SD) (C.) Percent of silencing in liver, kidney, quadriceps, and heart after SC injection of DCA siRNA targeting *Huntingtin* (*Htt*) mRNA (n=6 mice per group, 20 mg/kg). mRNA levels were measured using QuantiGene® (Affymetrix), normalized to a housekeeping gene, *Hprt* (Hypoxanthine-guanine phosphoribosyl transferase), and presented as percent of PBS (Phosphate buffered saline) control (mean ± SD). Data analysis: t test (***P<0.001, *P<0.1). ***Data in this figure are derived from (42).***

Tissue	Dose (mg/kg)	Level of accumulation (pmol of siRNA / mg of tissue)	Organ weight (mg)	% injected dose
Liver	20	28 ± 3	1000 (defined experimentally)	71 ± 8
	2*20	47 ± 4		59 ± 5
	6*20	81 ± 8		34 ± 3
	2*20 (1 month)	12 ± 1		15 ± 1
Heart	20	1.4 ± 0.1	94 (defined experimentally)	0.34 ± 0.030
	2*20	1.6 ± 0.2		0.19 ± 0.002
	6*20	1.7 ± 0.1		0.07 ± 0.003
	2*20 (1 month)	0.4 ± 0.04		0.04 ± 0.005
Full body muscles	20	0.4 ± 0.06	2400 (based on publications 53-55)	2.5 ± 0.4
	2*20	0.4 ± 0.04		1.2 ± 0.2
	6*20	0.9 ± 0.05		1.0 ± 0.1
	2*20 (1 month)	0.1 ± 0.03		0.4 ± 0.1

Fig. S4: Levels of tissue accumulation (pmol of siRNA / mg of tissue), and percent of the injected dose retained in liver, heart, and muscles after injection of various doses (20, 2*20 and 6*20 mg/kg), calculated using either experimentally defined organ weights (liver and heart) or based on literature derived organ weights (muscles). Accumulation levels were measured by PNA hybridization assay at 1-week or 1-month post-injection (average of n = 3 ± SD).

Cytokine (pg/mL)	PBS			DCA siRNA 50 mg/kg dose			DCA siRNA 100 mg/kg dose			Chol siRNA 50 mg/kg dose			Chol siRNA 100 mg/kg dose		
	1	2	3	1	2	3	1	2	3	1	2	3	1	2	3
IL-1alpha	13.0	12.6	12.2	12.8	13.4	12.2	15.0	13.4	13.0	12.8	14.6	14.6	19.0	22.1	25.1
IL-1beta	2.8	2.9	3.0	3.0	3.3	2.7	4.0	3.2	2.8	2.7	3.3	2.8	5.2	5.8	6.3
IL-2	3.4	3.5	3.5	8.5	3.5	13.5	4.1	6.8	3.8	3.5	4.0	4.3	5.6	6.0	6.5
IL-3	1.1	1.1	1.1	1.2	1.1	1.2	1.1	1.3	1.2	1.1	1.2	1.1	1.7	2.1	2.4
IL-4	3.1	3.2	3.3	3.3	3.4	3.1	3.9	3.4	3.3	3.3	3.9	3.4	5.9	7.2	8.6
IL-5	7.8	7.8	7.8	9.2	9.2	9.2	10.3	11.8	8.9	7.8	11.6	9.5	17.6	20.7	23.8
IL-6	10.4	10.4	10.4	11.3	11.5	11.0	15.3	17.9	12.7	10.4	15.0	13.3	31.7	31.6	31.4
IL-9	239.0	224.1	209.2	224.1	209.2	239.0	249.0	298.8	229.1	209.2	259.0	239.0	433.3	460.7	488.0
IL-10	18.3	17.5	16.7	17.8	19.3	16.2	23.0	18.3	17.8	17.8	21.4	18.8	31.3	37.4	43.4
IL-12p70	33.6	35.7	37.8	40.0	41.0	38.9	33.0	35.4	37.8	53.6	67.0	69.5	42.2	72.6	103.1
IL-13	5.2	5.0	4.8	5.1	5.0	5.2	5.4	7.3	5.0	5.2	6.3	5.9	10.0	11.6	13.1
IL-15/IL-15R	5.8	5.6	5.4	5.6	5.6	5.6	6.5	6.5	5.6	5.4	6.5	5.8	9.8	11.9	14.0
IL-17A	10.6	10.6	10.6	11.2	11.0	11.4	11.0	11.9	11.4	11.0	11.9	11.0	16.5	17.8	19.1
IL-18	163.2	155.1	146.9	168.7	168.7	168.7	195.9	223.1	179.5	179.5	223.1	195.9	375.4	432.5	489.7
IL-22	49.5	44.1	38.7	46.3	47.4	45.2	58.1	43.0	36.6	40.9	44.1	38.7	75.3	93.1	110.8
IL-23	62.5	61.2	59.8	63.2	65.3	61.2	87.0	59.8	57.1	59.8	62.5	57.1	89.7	107.4	125.1
IL-27	25.0	22.6	20.2	22.6	21.2	24.1	28.9	30.8	19.2	21.2	23.1	21.2	26.0	24.5	23.1
IL-28	129.9	122.1	114.3	128.6	122.1	135.1	142.9	124.7	124.7	129.9	135.1	129.9	223.5	278.1	332.6
IL-31	20.1	20.1	20.1	20.1	20.1	20.1	21.8	23.5	20.9	20.9	21.8	20.9	32.7	36.0	39.4
GM-CSF	4.1	4.0	3.9	3.9	4.1	3.8	4.4	4.6	4.1	4.1	4.4	4.4	7.5	8.6	9.6
M-CSF	0.9	0.9	0.9	1.0	1.0	1.0	0.9	1.4	1.1	1.0	1.2	1.1	2.0	2.4	2.9
G-CSF/CSF-3	8.8	8.9	9.1	9.5	9.8	9.1	11.2	11.9	10.5	9.5	18.9	10.5	61.2	64.9	68.7
IP-10	21.1	19.6	18.0	22.9	21.8	24.1	17.4	25.0	32.6	47.9	78.6	68.7	231.9	231.1	230.2
MCP-1	25.2	27.3	29.4	35.7	36.9	34.4	70.7	87.3	45.3	53.5	113.3	68.8	1363.9	2039.2	2714.4
MCP-3	18.0	19.0	20.0	82.6	88.6	76.5	78.0	105.2	132.3	153.4	333.4	206.8	469.5	473.0	476.5
MIP-1alpha	2.0	1.9	1.9	1.9	1.9	1.9	2.2	2.4	2.2	1.9	2.4	2.4	5.9	6.5	7.2
MIP-beta	3.0	3.0	3.0	3.1	3.0	3.2	4.2	5.4	4.6	4.2	5.3	5.8	38.4	36.3	34.3
MIP-2	3.5	3.5	3.5	3.9	3.9	4.0	3.6	5.3	3.8	3.9	4.7	6.5	6.8	7.3	7.9
Eotaxin	483.6	479.9	476.1	484.3	493.7	474.8	472.5	829.3	705.8	617.7	825.2	839.5	1181.0	1158.2	1135.4
RANTES	20.7	20.4	20.0	17.7	19.0	16.5	20.6	29.5	22.0	17.7	21.5	23.8	30.5	34.0	37.5
Gro-alpha/KC	19.2	17.3	15.4	20.0	25.1	14.9	34.4	53.1	15.6	21.8	30.8	17.5	65.9	69.4	73.0
IFN-alpha	25.6	28.0	30.5	34.4	43.0	25.7	35.9	33.9	31.8	23.1	35.7	21.1	83.3	96.5	109.7
IFN-gamma	3.3	3.2	3.1	3.3	3.1	3.4	3.5	4.0	3.4	3.3	3.6	3.4	4.7	5.4	6.1
TNF-alpha	10.7	11.0	11.2	11.0	10.3	11.7	23.3	26.6	13.1	11.7	17.7	11.0	30.4	29.7	29.0

Fig. S5: DCA conjugate does not induce cytokine elevation at both doses where cholesterol showed significant toxicity at high dose. Heat map showing cytokine concentrations (pg/mL) in serum at 24h after SC injections of DCA- and cholesterol (chol)-conjugated siRNAs at 50 and 100 mg/kg (n=3 mice per group, 1, 2 and 3 represent each mouse).

Cytokine (pg/mL)	PBS			DCA siRNA 20 mg/kg dose			DCA siRNA 2*20 mg/kg dose			DCA siRNA 6*20 mg/kg dose		
	1	2	3	1	2	3	1	2	3	1	2	3
G-CSF	10.9	12.4	13.3	9.9	12.6	12.8	10.9	9.3	10.7	10.7	11.4	12.8
GRO alpha	24.1	33.4	45.8	9.5	51.9	25.7	22.6	31.3	23.6	14.7	36.3	30.3
IFN gamma	6.0	5.8	6.0	5.3	5.7	6.2	5.9	5.8	5.3	6.2	6.1	6.8
IL-12p70	15.9	16.9	17.9	15.4	17.6	18.6	15.9	15.4	15.4	16.9	16.4	17.4
IL-6	15.5	15.5	17.9	14.4	19.1	19.1	15.5	15.5	15.5	15.5	16.7	19.1
IP-10	24.4	24.7	28.0	12.0	20.4	33.8	21.3	20.5	16.6	23.5	38.9	36.4
M-CSF	0.6	0.6	0.6	0.5	0.6	0.8	0.5	0.5	0.4	0.5	0.6	0.6
MCP-1	15.6	18.5	21.4	13.9	17.3	17.3	18.5	17.3	15.6	20.8	19.6	15.6
MCP-3	14.2	14.1	10.2	13.7	8.1	15.7	21.5	11.6	7.9	30.3	34.5	29.8
MIP-1beta	0.8	0.9	1.3	0.8	0.8	0.9	0.9	0.9	0.8	1.0	1.0	1.1
TNF alpha	10.3	11.0	14.1	7.5	10.8	9.7	9.1	8.8	8.1	10.3	8.1	10.3

Fig. S6: DCA conjugate does not induce cytokine elevation after multiple injections. Heat map showing cytokine concentrations (pg/mL) in serum at 1 week after SC injections of DCA-conjugated siRNAs at 20, 2*20, and 6*20 mg/kg (n=3 mice per group, 1, 2 and 3 represent each mouse).
